# The role of bacterial size, shape and surface in macrophage engulfment of uropathogenic *E. coli* cells

**DOI:** 10.1371/journal.ppat.1012458

**Published:** 2024-09-06

**Authors:** Elizabeth Peterson, Bill Söderström, Nienke Prins, Giang H. B. Le, Lauren E. Hartley-Tassell, Chris Evenhuis, Rasmus Birkholm Grønnemose, Thomas Emil Andersen, Jakob Møller-Jensen, Gregory Iosifidis, Iain G. Duggin, Bernadette Saunders, Elizabeth J. Harry, Amy L. Bottomley

**Affiliations:** 1 Australian Institute for Microbiology & Infection, University of Technology Sydney, Australia; 2 School of Life Sciences, University of Technology Sydney, Sydney, Australia; 3 Institute for Glycomics, Griffith University, Gold Coast, Australia; 4 Research Unit of Clinical Microbiology, University of Southern Denmark and Odense University Hospital, Odense, Denmark; 5 Department of Biochemistry and Molecular Biology, University of Southern Denmark, Odense, Denmark; University of Utah, UNITED STATES OF AMERICA

## Abstract

Uropathogenic *Escherichia coli* (UPEC) can undergo extensive filamentation in the host during acute urinary tract infections (UTIs). It has been hypothesised that this morphological plasticity allows bacteria to avoid host immune responses such as macrophage engulfment. However, it is still unclear what properties of filaments are important in macrophage-bacteria interactions. The aim of this work was to investigate the contribution of bacterial biophysical parameters, such as cell size and shape, and physiological parameters, such as cell surface and the environment, to macrophage engulfment efficiency. Viable, reversible filaments of known lengths and volumes were produced in the UPEC strain UTI89 using a variety of methods, including exposure to cell-wall targeting antibiotics, genetic manipulation and isolation from an *in vitro* human bladder cell model. Quantification of the engulfment ability of macrophages using gentamicin-protection assays and fluorescence microscopy demonstrated that the ability of filaments to avoid macrophage engulfment is dependent on a combination of size (length and volume), shape, cell surface and external environmental factors. UTI89 filamentation and macrophage engulfment efficiency were also found to occur independently of the SOS-inducible filamentation genes, *sulA* and *ymfM* in both *in vivo* and *in vitro* models of infection. Compared to filaments formed via antibiotic inhibition of division, the infection-derived filaments were preferentially targeted by macrophages. With several strains of UPEC now resistant to current antibiotics, our work identifies the importance of bacterial physiological and morphological states during infection.

## Author summary

Urinary tract infections (UTIs) are one of the most common bacterial infections worldwide with 50% of women suffering from a UTI during their lifetime. *Escherichia coli* is the primary bacteria responsible for UTIs and is usually found in short rod forms. However, during UTIs *E. coli* can elongate into extremely long thin shapes called ‘filaments’. Filaments are thought to be advantageous during infections because they are too long to be engulfed and killed by immune cells called macrophages. Due to increasing antibiotic resistance in bacteria there is a strong need for the discovery of new ways to treat infections and this is only possible once we thoroughly understand the mechanisms bacteria employ to overcome our immune response. Therefore, we investigated the effect of *E. coli* filamentation on macrophage engulfment along with other aspects of bacteria reported to influence engulfment. We found that the ability of filaments to avoid macrophage engulfment is dependent on a combination of size (length and volume), shape, surface and external environmental factors. Our research has highlighted the importance of bacterial shape changes during infections and provided a foundational understanding of macrophage engulfment of filaments. Eventually, this knowledge may reveal new targets for treatment of infections.

## Introduction

The phenomenon of rod-shaped bacteria to change shape into long “spaghetti-like” filamentous cells has been repeatedly observed in a variety of species [1,2] under many different environmental conditions [3]. Filaments arise when cell division is inhibited whilst DNA replication and chromosome segregation continue, with filaments containing multiple copies of the chromosome along their lengths [2,4,5]. Whilst this morphology has been commonly observed, the biological impact of filamentation is still largely unknown, although it has been suggested to provide survival advantages in various niches, such as protection against aquatic protist predators for *Flectobacillus* species [6], host intracellular survival for *Burkholderia pseudomallei* [7] or protection against phagocytosis by host immune cells during infection [8,9]. Understanding why bacteria filament in certain environments will provide insights into their pathogenicity and the potential of this morphology to impede the treatment of infections, particularly those caused by antibiotic resistant bacteria [4,10–12].

Urinary tract infections (UTIs) are extremely common, affecting around 150 million people globally every year, with uropathogenic *Escherichia coli* (UPEC) causing over 80% of these infections [13,14]. The only treatments available for UTIs are antibiotics and with several UPEC strains currently resistant to many antibiotics, they are an increasing threat to human health [15]. UPEC can undergo extensive filamentation during acute UTIs as part of the infection cycle [16,17] though the exact role of this morphological change during the infection cycle is not conclusive [16,18]. Compared to their rod counterparts, filaments may more strongly adhere to vulnerable host cells, and consequently be more effective at establishing infection [19], and/or be better able to avoid phagocytosis by immune cells and thus subvert host immunity during infection [8,9]. Identifying new treatment approaches should be possible with a detailed understanding of the role(s) that bacterial morphology of pathogenic bacteria, such as UPEC, in interacting with the host immune system during infection.

UPEC filaments are not efficiently engulfed and killed by neutrophils and macrophages in a mouse model of acute UTI [8,11,20]. It has been hypothesised that the increase in bacterial cell size prevents immune cells effectively engulfing and clearing the infection [21,22]. However, immune cells can engulf large particles, such as apoptotic or cancerous tissue cells, clumps of bacteria or inorganic foreign bodies up to 20 μm long [23,24], leading to the proposal that it is more than just bacterial cell size that results in filaments being engulfed less readily than shorter, rod-shaped cells [20]. Computational modelling and biophysical studies using silica or polystyrene nanoparticle beads have examined the interdependence of shape and size in phagocytosis efficiency, showing both aspect ratio and curvature contribute to the ability of immune cells to efficiently phagocytose; prolate particles are more difficult to engulf because of their high aspect ratios and rounded tips [24,25]. However, how this interplay between size and shape to prevent immune cell engulfment translates to changes in bacterial morphology in live pathogenic bacteria is still relatively unknown.

Understanding the role of filamentation in avoiding immune cell engulfment, and what biophysical aspects contribute to this avoidance, is further complicated by the significant variation in experimental methods used to generate filaments. These variations include stimuli to induce filamentation, bacterial cell viability, bacterial species, use of inert particles and immune models, making it difficult to draw definitive conclusions about what aspect(s) of bacterial filamentation provides a survival advantage [22,26,27]. Even the fundamental definition of what constitutes a filament is not consistent, with ‘filaments’ varying from 5 μm to over 200 μm depending on the induction method, and the definition of filament length is inconsistently reported between studies [9,16,17,28]. A complete understanding of the role and significance of filamentation, in the context of UPEC and the implications for UTIs, requires a rigorous exploration of what properties of filaments are important in affecting macrophage engulfment.

Here, we have used controlled conditions to generate UPEC filaments of defined length, allowing us to unravel the importance of biophysical parameters such as bacterial cell size and shape, and physiological parameters such as cell surface and the environment, during engulfment by human macrophages. Using gentamicin-protection assays and fluorescence microscopy to quantify the engulfment ability of macrophages, we found that UPEC filaments derived from all conditions tested were engulfed less by macrophages compared to their rod counterparts. There was an interplay between size and shape of bacterial cells, where spherical cells were more readily engulfed compared to both rods and filaments. Mannose-dependent interaction between UPEC and human macrophages was confirmed as the major pathway for engulfment, regardless of bacterial cell size. Decreased complete engulfment of UPEC filaments isolated from an *in vitro* bladder model was also observed. However, an increase in partial engulfment of bladder model filaments compared to antibiotic-induced filaments demonstrated an influence of the bacterial growth environment on engulfment effectiveness. Finally, deletion of the SOS response genes *sulA* and *ymfM* had no effect on the ability of UPEC to filament or influence UPEC engulfment by macrophages. We conclude from these observations that while the size and shape of UPEC are major influencers of effective macrophage engulfment, the growth environment of UPEC rods and filaments also plays a role in macrophage engulfment.

## Results

### Cephalexin treatment results in a population of viable UTI89 filaments of defined length

**Fig 1:**
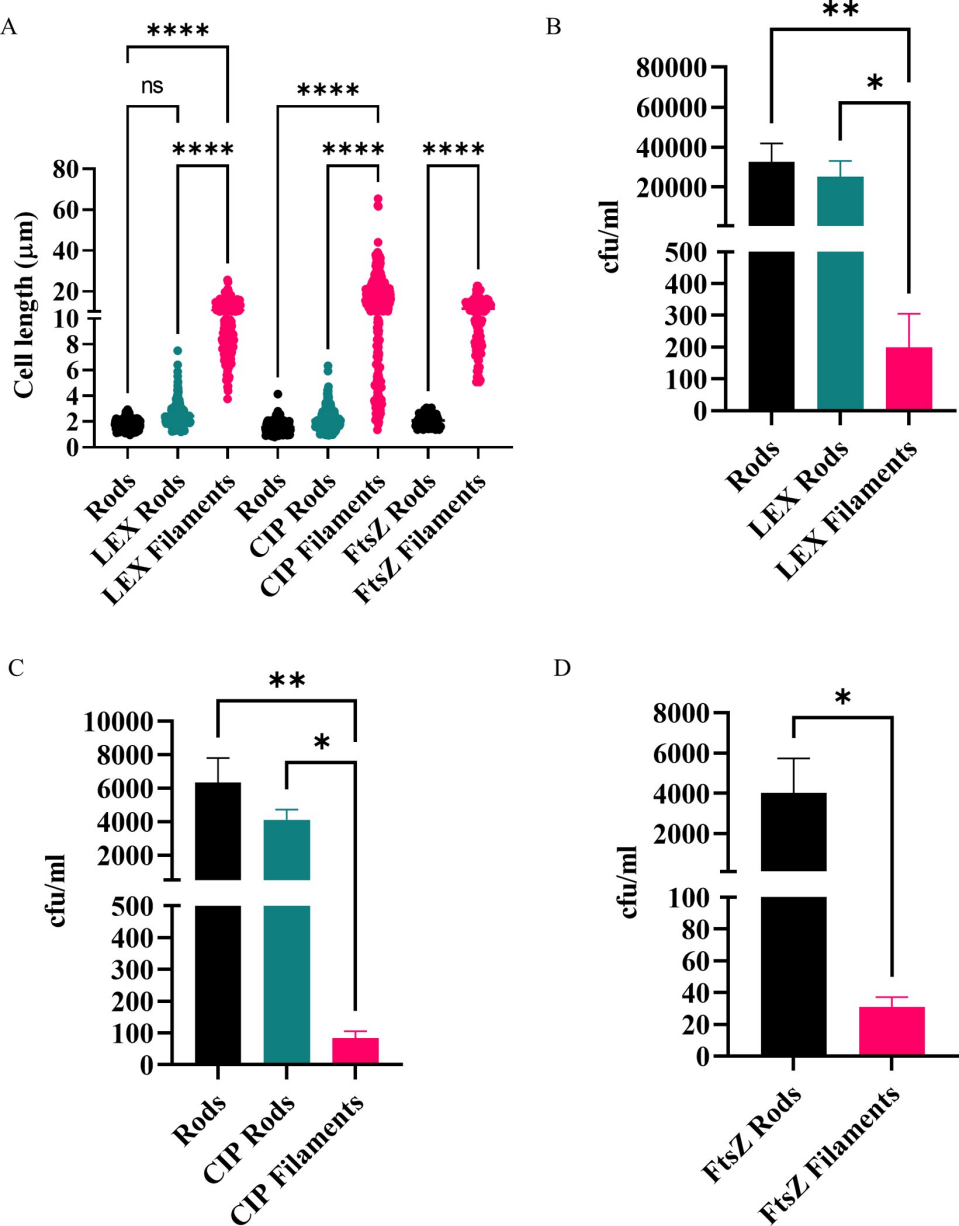
THP-1 macrophages engulf UTI89 filaments significantly less than rods. UTI89 was treated with either cephalexin (rods: untreated, LEX rods: 2.5 μg/ml, LEX filaments: 10 μg/ml) or ciprofloxacin (rods: untreated, CIP rods: 3.75 ng/ml, CIP filaments: 15 ng/ml). UTI89/pLau80 was treated with 0.2% glucose (FtsZ (plasmid repressed) rods) or 0.2% arabinose to induce expression of ftsZ-yfp (FtsZ-overproduction filaments). (A) Bacterial lengths as determined by phase contrast microscopy with n = 110–426. (B-D) THP-1 macrophages were infected (MOI 10) for 1 hour and bacterial loads were assessed 2-hours post infection in a gentamicin-protection assay. Data are the averages of 4 independent experiments with error bars representing the SEM. * indicates p <0.05, ** p <0.01, **** p <0.0001, determined by one-way ANOVA with multiple comparisons or Welch’s t-test.

We initially produced viable *E. coli* UTI89 filaments of defined length using cephalexin since it targets PBP3/FtsI to block cell division [29,30] and is used in the treatment of UTIs [31,32]. The minimum inhibitory concentration (MIC) of cephalexin in UTI89 was determined to be 5 μg/ml and, to induce filamentation, antibiotic concentrations used were the same as the range detected in the plasma of healthy individuals who have undergone antibiotic treatment (7.7–12.3 μg/ml [32]). At 10 μg/ml, cephalexin-induced filaments had a mean bacterial cell length of 10 μm (± 0.3 SEM, population range 3.8–25.5 μm) and were 6-fold longer than untreated rods (1.7 μm ± 0.02 SEM; population range of 1.0–2.9 μm; Figs 1A, AA, and AB in S1 Text). To control for the effect of antibiotic exposure which may affect macrophage engulfment but is unrelated to bacterial cell length changes, UTI89 was exposed to a low (sub-MIC) concentration of cephalexin (2.5 μg/ml). These bacterial cells had a mean cell length of 2.4 μm (± 0.06 SEM; population range of 1.2–7.5 μm Fig 1A) and were not statistically significantly different in length to untreated rods. Bacteria from all three populations were viable as measured by membrane permeability and ATP level assays [18], and cephalexin-induced filamentation was reversible; removal of cephalexin resulted in the resumption of cell division, within 1 hour, producing an almost uniform population of rods by 2 hours incubation in LB media (Figs DA and DD in S1 Text). For the purpose of this study, we defined rods as less than 4 μm long, to account for a doubling in the average size of an *E*.*coli* rod (2 μm) before a division event. On average, filaments within this study were at least 6-fold longer than rods. For quantification purposes bacterial cells were classified as rods (less than 4 μm), intermediate (4–10 μm), and filaments (over 10 μm) to allow clear differentiation between filaments and its rod counterpart.

### Cephalexin- and ciprofloxacin-induced filamentation of UTI89 protects against macrophage engulfment

The THP-1 human monocyte cell line (ATCC TIB-202), differentiated with phorbol-12-myristate-13-acetate (PMA) to form macrophages, was used to model phagocytic engulfment of cephalexin-induced UTI89 filaments and untreated rods in a gentamicin-protection assay. A gentamicin-protection assay with an infection time of 1 hour and a gentamicin kill time of 1 hour was used in this study following previously published methods [33]. A multiplicity of infection (MOI) of 10 was used as for other UPEC engulfment studies [33–35]. We established that only a small proportion of the cephalexin-induced filament population reverts to rods in the gentamicin protection assay growth media within the time frame of the assay (Fig E in S1 Text).

When performing gentamicin protection assays, it was critical that we were able to directly compare the colony forming units per ml (cfu/ml) values for rods and filaments given that they differ in their mass. Therefore, we first examined the relationship between cfu/ml and absorbance (bacterial biomass) to confirm that one filament, under the conditions of our experiments, resulted in one cfu. Cephalexin treatment resulted in filaments with a mean length 6.1-fold that of rods. We found, by measuring the absorbance and cfu/ml of these populations over time, that the biomass (as measured by absorbance at 600 nm) of populations increases steadily for all populations, although addition of 10 μg/ml cephalexin slightly reduced the growth rate (Fig FA in S1 Text). After the addition of 10 μg/ml cephalexin there is little increase in cfu/ml of filaments (Fig FB in S1 Text), as expected due to a block in cell division whilst cells continue to grow. The cfu/ml of rods was approximately 6-fold greater than that of filaments (Fig FB in S1 Text), consistent with the ~6-fold increase in filament length over the same time (Fig A in S1 text). From these data, we can conclude that ~1 cfu corresponds to ~1 bacterium (rod or filament), and filaments represent an equivalent biomass of ~6 rods under these experimental conditions. This validates the comparisons of rod and filament engulfment measured by cfu/ml in the gentamycin-protection assays.

Gentamicin-protection assays revealed that cephalexin-induced filaments (LEX filaments) were engulfed by macrophages 150-fold less frequently than untreated rods (Fig 1B; p = 0.009). There was no significant change to macrophage engulfment levels between low dose cephalexin-exposed rods (2.5 μg/ml; LEX rods) and untreated rods (Fig 1B), suggesting that the reduction of ability of macrophages to engulf filaments is at least in part a consequence of the morphological change of filamentation caused by antibiotic treatment, rather than just antibiotic exposure *per se* (see also FtsZ-YFP expression induced-filaments below).

We next examined whether induction of UTI89 filamentation, using an antibiotic that has a different target to PBP3 but is also used in the treatment of UTIs [36], leads to a similar decrease in macrophage engulfment. Bacteria treated with 15 ng/ml ciprofloxacin, which targets DNA gyrase [37], resulted in viable, reversible filamentation in which bacteria were 10-fold longer than both untreated and low-dose ciprofloxacin-treated (3.75 ng/ml) control rods (Figs 1A, AC-AD, BB, CB, and DB in S1 Text). As with cephalexin-induced filamentation, long bacteria produced by ciprofloxacin treatment of UTI89 (CIP filaments, population range of 1.4–65.4 μm) provided significant protection against macrophage engulfment; being engulfed 60-fold less than their rod counterpart (Fig 1C). As observed for LEX rods, low-dose ciprofloxacin treated rods (CIP rods; population range of 1.0–6.3 μm) were similarly engulfed compared to untreated UTI89 (Fig 1C). Overall, these results indicate that filamentation provides an advantage during macrophage engulfment, regardless of the antibiotic stimuli to induce filamentation.

### UTI89 filamentation induced by abnormal FtsZ expression also protects against engulfment by macrophages

We found that exposure to low doses of antibiotic did not alter macrophage engulfment compared to untreated bacteria, suggesting that it is increased cell length, and not antibiotic treatment, that results in drastically low engulfment of filaments. To confirm this, we used a stimulus that did not rely on antibiotics to induce filamentation. FtsZ was produced (as a YFP fusion) from pLau80 [38] under P_BAD_ control, in addition to endogenous FtsZ in UTI89. This induced strong filamentation [39] on addition of arabinose (herein referred to as FtsZ-overproduction filaments) [40]. FtsZ -overproduction filaments were 5.5-fold longer than their rod counterpart (11.4 μm ± 0.4 SEM, population range of 5.1–22.6 μm vs. FtsZ (plasmid repressed) rods; 2.1 μm ± 0.04 SEM; population range of 1.4–3.1 μm Figs 1A and AE-F in S1 Text) and were as viable as FtsZ (plasmid repressed) rods (Fig BC in S1 Text) but showed a moderate decrease (29%, *p* = 0.002) in ATP production compared to FtsZ (plasmid repressed) rods (Fig CC in S1 Text). This could be due to the overabundance of FtsZ (as a combination of FtsZ and FtsZ-YFP) affecting metabolism when FtsZ levels are usually tightly controlled [41]. As with the antibiotic-induced filaments, filamentation was reversible once *ftsZ-yfp* expression was repressed (Fig DC in S1 Text), albeit taking a longer time to revert to rods than the removal of antibiotics (by 2 hours over 50% of bacteria remained over 4 μm in length), presumably due to the need to reduce the level of FtsZ-YFP appropriately. Macrophage engulfment of FtsZ-overproduction filaments was drastically decreased (100-fold) compared to FtsZ (plasmid repressed) rods (Fig 1D), showing that non-antibiotic induced filamentation also provides substantial protection against macrophage engulfment. The combined results above demonstrate that the actual length of the UTI89 filament is at least one key factor in providing this protection.

### Rods are preferentially engulfed by macrophages in mixed populations of rods and filaments

**Fig 2:**
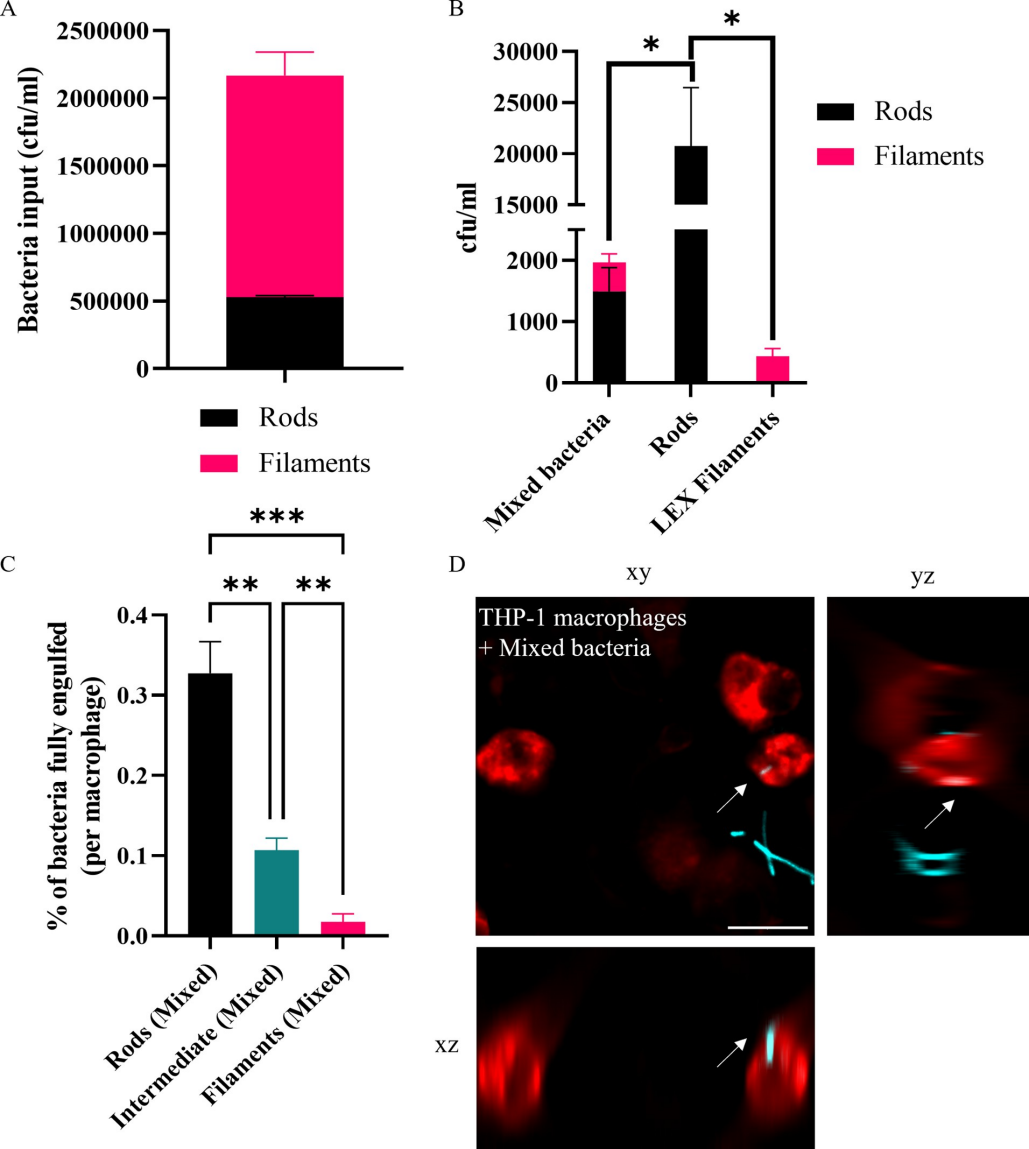
In a heterogeneous population of rods and filaments, rods are engulfed by THP-1 macrophages preferentially over filaments. UTI89 and UTI89/pGI5 (msfGFP) were untreated (rods) or exposed to 10 μg/ml cephalexin (LEX filaments). (A) rods and filaments were combined to create one population (Mixed bacteria) of 24% rods and 76% filaments. (B) THP-1 macrophages were infected (MOI 10) for 1 hour and intracellular bacterial loads were assessed 2-hours post infection in a gentamicin-protection assay. (C-D) Macrophages were infected with UTI89/pGI5 (msfGFP) (cyan) Mixed bacteria at MOI 10, fixed after 60 minutes and stained with 1X CellMask Orange (red). Images were acquired using a DeltaVision Elite microscope with the 40X dry NA 0.60 objective. (C) Percentage of bacterial populations fully engulfed (number of bacteria fully engulfed/total number of bacteria counted X 100) is normalised and presented as per macrophage to allow for comparisons despite variations in number of macrophages counted from microscopy images. (D) Representative image of infected macrophage with arrow indicating an internalised rod bacterium. Orthogonal xz and yz views highlight internalisation of rod bacterium. Scale bar = 20 μm. Data are the averages of 4 independent experiments (A and B) or 2 independent experiments (C) with error bars representing the SEM. * indicates p < 0.05, ** p < 0.01, determined by one-way ANOVA with multiple comparisons or Welch’s t-test.

We have shown that upon various stimuli, viable filaments are engulfed by macrophages significantly less than rods. However, this is for homogenous rod or filament populations. A mixed population of rods and filaments is the norm in most environments [17,42,43] which may influence the engulfment dynamics of macrophages. We therefore investigated macrophage engulfment of a mixed rod and filament UTI89 population using cephalexin to stimulate filament formation since this treatment resulted in a reproducible mean filament length. We used strains UTI89 and UTI89/pGI5 (the latter constitutively producing monomeric superfolder GFP, msfGFP, from pGI5 [44]) to produce separate populations of viable rods and msfGFP-labelled filaments which were subsequently combined to produce a mixed population of rods (1.6 μm ± 0.03 SEM) and filaments (10.3 μm ± 0.2 SEM). A UTI bladder infection model is comprised of approximately 80% UTI89 filaments (measured via microscopy) [45], and to mimic this, we created a heterogeneous population of approximately 76% filaments and 24% rods, based on known CFU/ml data (Fig 2A). Using a gentamicin-protection assay with the mixed population of UTI89 and UTI89/pGI5 (msfGFP) bacteria, macrophage engulfment was 10-fold less than a UTI89 rod-only population (Fig 2B), indicating reduced overall engulfment in a heterogeneous population. However, while only 24% of the mixed population added to macrophages was rods, 76% of the plate-recovered engulfed bacteria consisted of rods as determined by selective antibiotic plating (due to spectinomycin resistance from pGI5 (msfGFP); Fig 2A and 2B), indicating that there is also a distinct preference for engulfment of rods over filaments in a heterogeneous mixed population. The plasmid had no effect on engulfment, and it made no difference which population (rods or filaments) contained the plasmid (Fig G in S1 Text).

To directly visualize the interaction between THP-1 macrophages and bacteria, widefield fluorescence microscopy was performed following a 60-minute incubation with a mixed population of UTI89/pGI5 (msfGFP) rods and filaments. Cell lengths of bacteria were measured and showed that rods (bacteria < 4 μm in length, to account for bacteria preparing to divide) were engulfed preferentially over filaments (bacteria > 10 μm in length) after a 1-hour infection (Fig 2C–2D). Microscopy analysis of fully engulfed bacteria (bacteria completely enveloped by the macrophage membrane) showed that in the artificial mixed population the rod populations were fully engulfed significantly more than filaments. Cells of intermediate length (4–10 μm) were fully engulfed more than rods, but significantly less than filaments. Approximately 0.33% of the rod populations added were fully engulfed by each macrophage, compared to less than 0.01% of the filament population (Fig 2C). Therefore, our data shows that as with separate populations, rods are engulfed preferentially over filaments in a mixed population.

### As bacterial cell length increases macrophage engulfment decreases at an exponential rate

**Fig 3:**
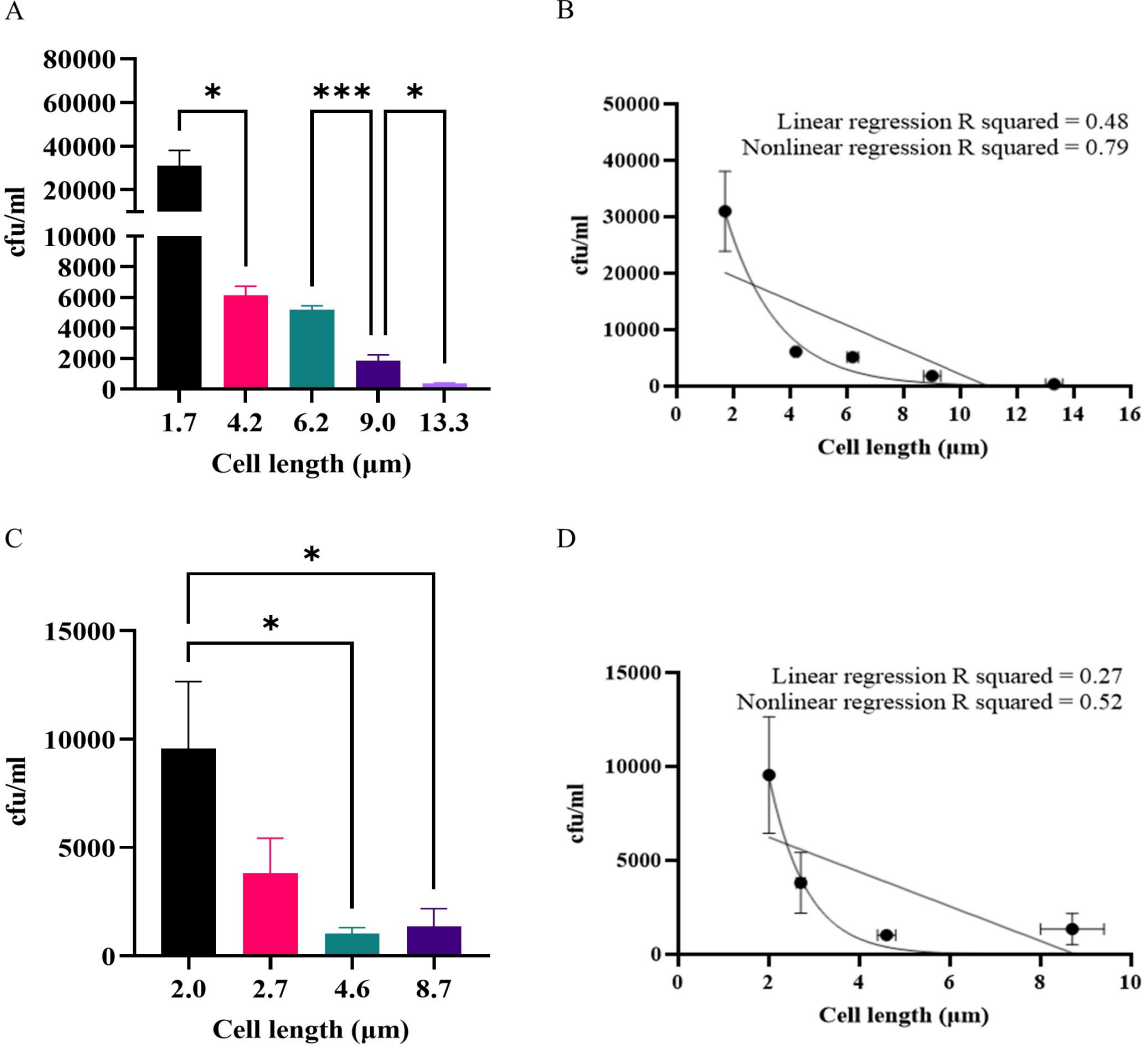
THP-1 macrophage engulfment of UTI89 decreases exponentially as bacterial length increases. (A-B) UTI89 was treated with 10 μg/ml cephalexin for increasing periods of time to produce filaments of increasing average length. (C-D) UTI89/pLau80 was treated with 0.2% (v/v) arabinose for increasing periods of time to produce FtsZ filaments of increasing average length. (A-D) THP-1 macrophages were infected (MOI 10) for 1 hour and bacterial loads were assessed 2-hours post infection in a gentamicin-protection assay. Data are the averages of 4 independent experiments with vertical error bars representing the SEM of intracellular bacteria and horizontal error bars (B and D) represent SEM of bacterial length, n = 105–250. * indicates p < 0.05, ** p < 0.01, *** p < 0.001, determined by one-way ANOVA with multiple comparisons. R squared values generated from linear and nonlinear regression analyses, which were plotted as straight or curved lines, respectively, on B and D.

Previous studies, primarily examining non-viable particles, have demonstrated that the optimal particle size for engulfment by macrophages is between 1 to 3 μm [26,46–48]. However, macrophages have been reported to engulf very large particles, such as latex beads or apoptotic mammalian cells over 20 μm in diameter [27,49]. We therefore sought to establish how long a bacterial cell needs to be before there is an observable reduction in macrophage engulfment. Bacterial cell populations of UTI89 with increasing average lengths were produced by staggering the time of addition of cephalexin (10 μg/ml) to produce populations varying in length from 1.7 to 13.3 μm (Fig 3A). Using a gentamicin-protection assay, we found that just doubling the bacterial cell length i.e., blocking the first division, from 1.7 μm for untreated cells to 4.2 μm with cephalexin exposure, is enough to decrease macrophage engulfment 5-fold (*p* = 0.039) (Fig 3A). This was surprising since it has been observed that UPEC filaments can get extremely long [18]. Additionally, we found that increasing bacterial cell length resulted in an exponential decrease in engulfment, (nonlinear regression R squared value = 0.79, Fig 3B). Similar trends were observed with filaments of varying lengths (between 2 and 8.7 μm) as a result of *ftsZ* expression (using UTI89/pLau80/pGI5 (*ftsZ-yfp*, msfGFP) cultures) (Fig 3C and 3D). Overall, while only a small increase in bacterial cell length (2.4-fold) is enough to cause a significant reduction in macrophage engulfment, continued increase in bacterial cell length results in a corresponding reduction in engulfment efficiency. Thus, there does not appear to be a length threshold that bacteria reach which suddenly decreases engulfment. A relatively small increase in bacterial cell length is enough to reduce macrophage engulfment and as length increases, engulfment continues to decrease at an exponential rate.

### Macrophage engulfment is influenced by both shape and size of UTI89 cells

**Fig 4:**
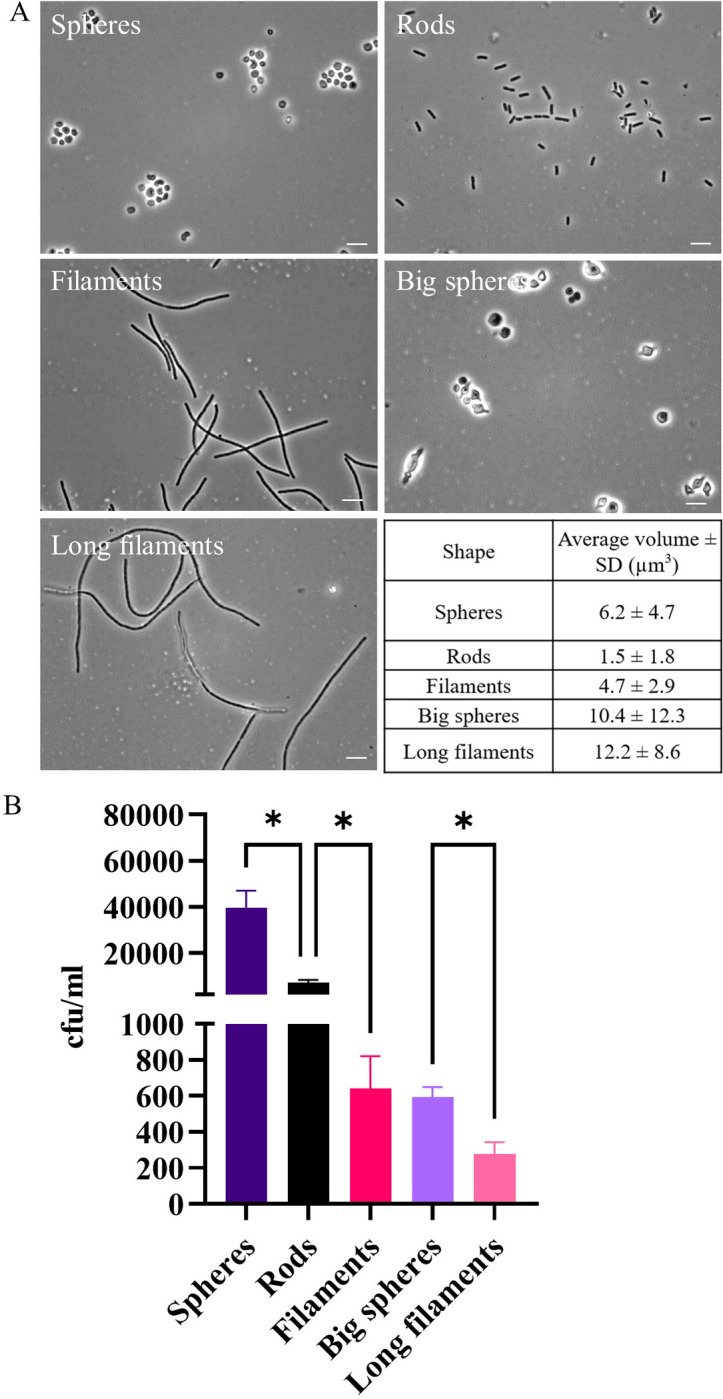
THP-1 macrophages have bacterial size and shape preferences during engulfment. UTI89 cells were untreated (rods) or treated with 10 μg/ml cephalexin (filaments), 10 μg/ml mecillinam (Spheres), and grown for 3 additional hours with 10 μg/ml mecillinam and cephalexin (Big spheres) or with 10 μg/ml cephalexin (Long filaments). (A) Phase contrast images were acquired using a Zeiss Axioplan 2 microscope with the 100X oil immersion NA 1.4 objective. Images are representative from 1 experiment. Scale bar = 5 μm. Table of average cell volumes with standard deviation (SD) as measured by a Coulter counter, n = 36927–281142. (B) THP-1 macrophages were infected (MOI 10) for 1 hour and bacterial loads were assessed 2 hours post infection in a gentamicin-protection assay. Data are the averages of 3 independent experiments with error bars representing the SEM. * indicates p < 0.05, determined by one-way ANOVA with multiple comparisons.

Previous studies using small inert polystyrene particles have indicated that it is the target shape rather than size that influences macrophage engulfment [26,50]. To establish whether this holds true for live bacterial cells, we created distinct shapes of UTI89 cells using two cell wall-targeting antibiotics. Cephalexin targets PBP3 (FtsI) to inhibit septal cell wall synthesis, resulting in filamentation [30,51], whilst mecillinam targets PBP2 to inhibit lateral cell wall synthesis, resulting in spherical bacterial cells [52–54]. By varying the time of exposure to individual antibiotics, or by combining treatments, bacterial cells with different sizes and shapes could be produced (Fig 4A). Cell volumes were measured to be able to compare the size of bacterial cells of different shapes.

Although untreated rods had the smallest mean bacterial cell volume of 1.6 μm^3^, we found that spherical mecillinam-treated UTI89 with a mean bacterial cell volume of 6.6 μm^3^, were engulfed 4-fold more than rods (Fig 4B), indicating that there is primarily a shape preference during engulfment. This is further demonstrated by comparing bacterial cells of different shape but similar volume: Spheres (6.6 μm^3^) were engulfed 62-fold more than filaments (4.7 μm^3^; Fig 4B). This shape dependency is conserved when bacterial cell volume is increased; Big spheres (10.3 μm^3^), produced by treatment with both antibiotics, were engulfed 2.5-fold more than Long filaments (11.1 μm^3^, Fig 4B). However, there is also a size dependency for engulfment, as increasing the size of spherical bacterial cells to Big spheres (10.3 μm^3^) decreased engulfment 80-fold compared to Spheres (6.6 μm^3^). This indicates that there is an interplay between size and shape of UTI89 cells that influences the effectiveness of macrophage engulfment.

### The reduced macrophage engulfment of filaments is unlikely to be due to changes in their ability to bind mannose

Since the bacterial cell surface is the initial point of recognition between a macrophage and its target [55], we investigated if surface glycans differed between cephalexin-induced rods and filaments which could account for differences in engulfment efficiency. Glycan microarray analysis revealed differences in the ability of rods, LEX rods and LEX filaments to bind to different glycan moieties (Table A in S1 Text). However, these differences did not appear to involve glycans known to be involved in influencing macrophage engulfment and are not predicted to be a major contributing factor in reduced engulfment of filaments [56].

**Fig 5:**
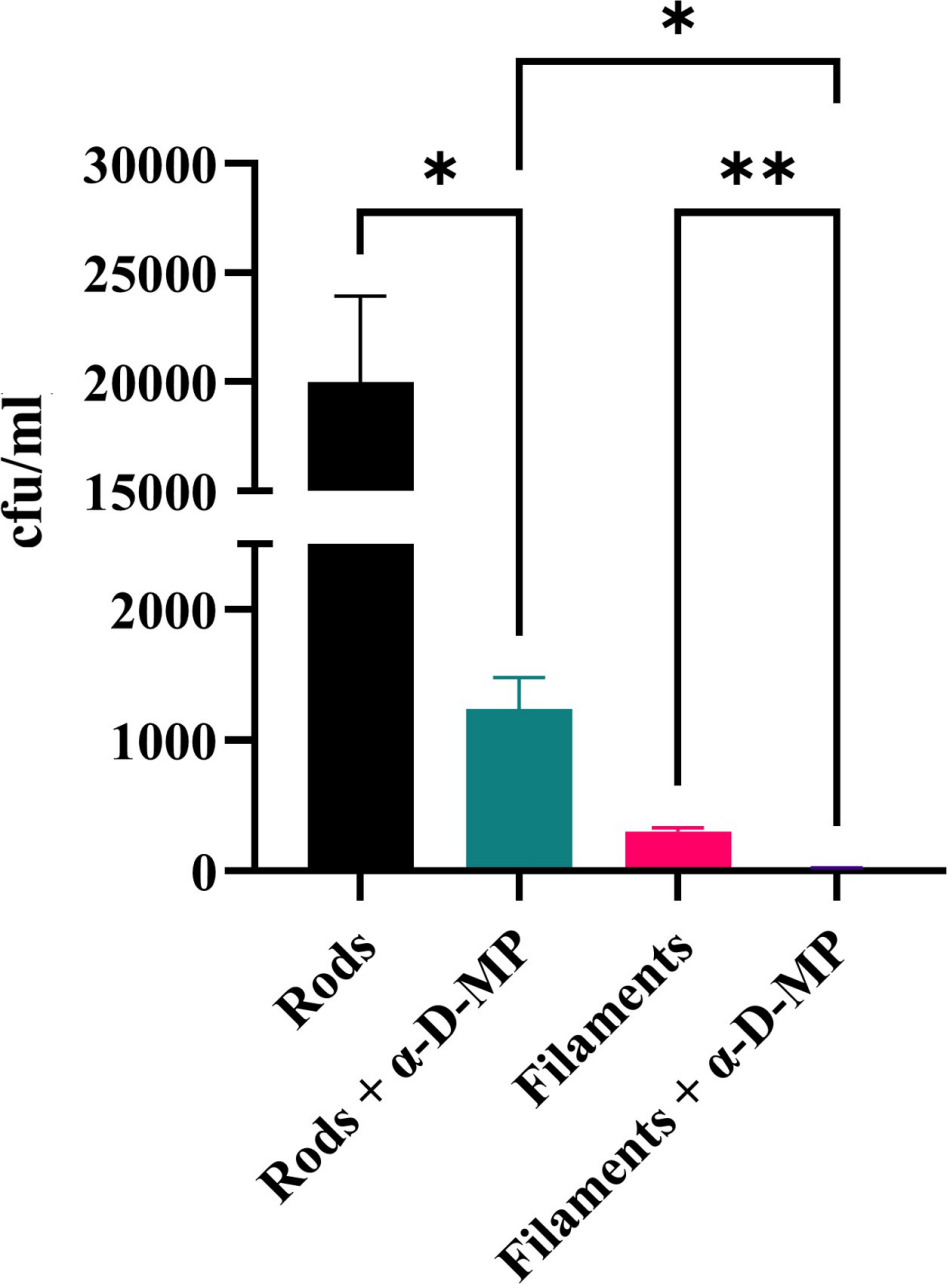
Blocking mannose binding with methyl α-D-mannopyranoside reduces THP-1 macrophage engulfment of UTI89 rods and filaments. UTI89 was untreated (rods) or treated with 10 μg/ml cephalexin (filaments). Both populations were also treated with 3% methyl α-D-mannopyranoside (rods + α-D-MP and filaments + α-D-MP). THP-1 macrophages were infected (MOI 10) for 1 hour and bacterial loads were assessed 2 hours post infection in a gentamicin-protection assay. Data are the averages of 3 independent experiments with error bars representing the SEM. * indicates p < 0.05, ** p < 0.01, determined by one-way ANOVA with multiple comparisons.

A well-characterised primary interaction between UPEC and macrophages is via mannosylated glycoproteins on the macrophage surface [57]. This binding can be competitively inhibited by methyl α-D-mannopyranoside (α-D-MP) [58], which binds to bacterial surface structures, thus blocking or reducing macrophage binding. Indeed it has previously been shown that addition of α-D-MP results in reduced mouse macrophage engulfment of *E. coli* J96 rods [58], demonstrating that engulfment of UPEC is mannose dependent. It is possible that filament engulfment is less efficient than that of rods due to reduced binding of filaments to host mannosylated receptors. If this were the case, then blocking mannose-binding would not lead to a significant decrease in filament engulfment but would decrease rod engulfment. We therefore tested this by adding α-D-MP to UTI89 rods and cephalexin-induced filaments (~10 μm length) and performing a gentamicin-protection assay with THP-1 macrophages. Consistent with previous results [58], engulfment of rods treated with α-D-MP decreased 16-fold compared to the untreated rod population in the presence of α-D-MP (*p* = 0.041; Fig 5). We observed a similar fold-decrease (13-fold, *p* = 0.009) in engulfment of filaments in the presence of α-D-MP relative to untreated filaments (Fig 5). Rods + α-D-MP were still engulfed significantly more (*p* = 0.038) than filaments + α-D-MP demonstrating that the preference for rods remained. The equal proportional decrease in engulfment of both rods and filaments when mannose binding is inhibited indicates that under these conditions the vast difference in THP-1 engulfment of filaments compared to rods is unlikely to be due to changes in their mannose-specific binding. It is also interesting to note that whilst engulfment of rods and filaments is significantly reduced, there is still a proportion of bacteria that are successfully engulfed when mannosylated glycoproteins are competitively inhibited (rods and filaments + α-D-MP), suggesting that there are mannose-independent interactions between UPEC and macrophages that are critical for optimal engulfment.

### Bacterial growth environment contributes to macrophage engulfment dynamics

We have established that UTI89 cell size and shape both contribute to the effectiveness of macrophage engulfment under certain conditions. Other factors such as the environmental conditions under which bacteria are grown could influence these factors. Whilst *E. coli* forms filaments in response to certain antibiotics, UPEC filaments have also been observed in the urine of patients with UTIs who have not undergone antibiotic treatment [17]. Furthermore, UTI89 forms filaments inside human bladder cells *in vitro* when induced by the flow of concentrated urine [18,28,44]. We therefore examined the ability of THP-1 macrophages to engulf UTI89 isolated from an *in vitro* human bladder model which has not been exposed to antibiotic treatment. This replicates a heterogeneous bacterial population of varying cell lengths, from very short cells to very long, and such populations have been observed in UTIs of mice and humans [17,44,45,59].

**Fig 6:**
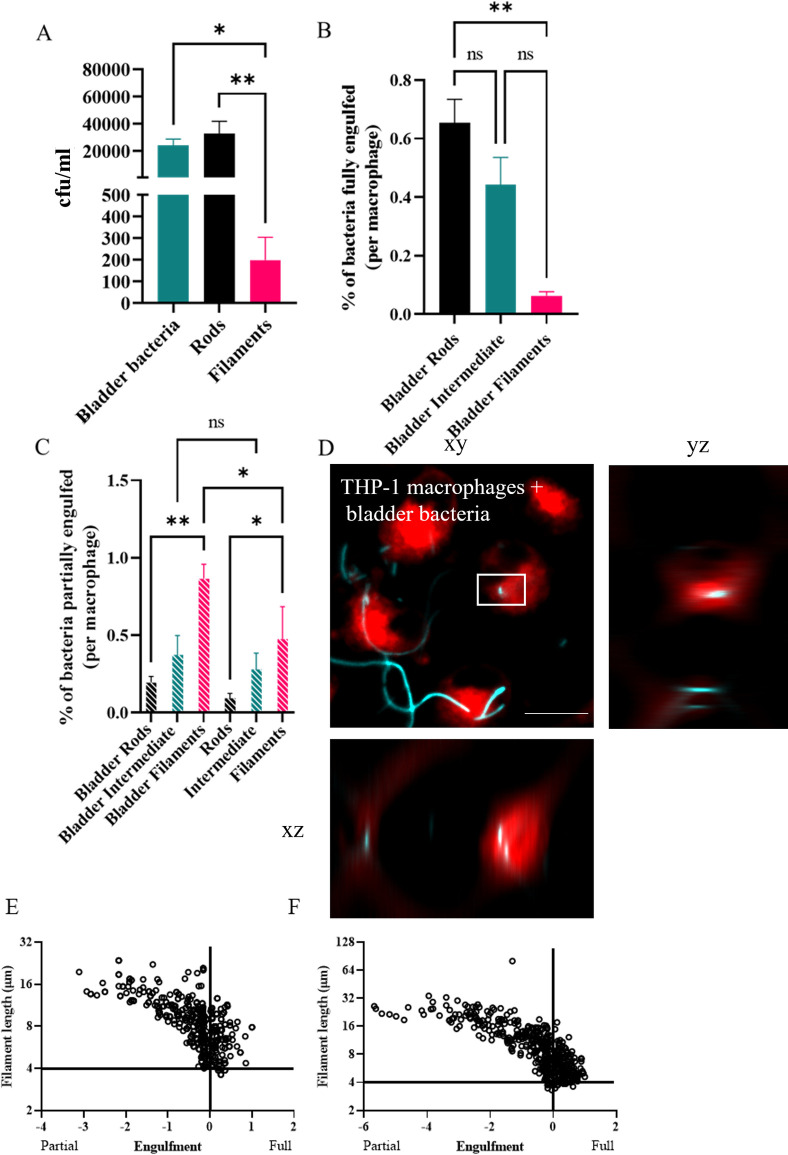
The growth environment of bacteria contributes to engulfment dynamics. UTI89/pGI5 (msfGFP) were isolated from an in vitro human bladder model (bladder bacteria; ~86% filaments) or treated with 10 μg/ml cephalexin (filaments) or untreated (rods) and grown in LB medium. (A) THP-1 macrophages were infected (MOI 10) for 1 hour and bacterial loads were assessed 2-hours post infection in a gentamicin-protection assay. (B-D) Macrophages were infected with bladder bacteria (cyan) at MOI 10, fixed after 60 minutes and stained with 1X CellMask Orange (red). Images were acquired using a DeltaVision Elite microscope with the 40X dry NA 0.60 objective. Internalisation of bacteria was determined using a FIJI macro on 3D image data. (B) Percentage of bacterial populations fully engulfed (number of bacteria fully engulfed/total number of bacteria counted) is normalised and presented as per macrophage to allow for comparisons despite variations in number of macrophages counted from microscopy images. (C) Percentage of bacterial populations partially engulfed per macrophage as determined by microscopy presented as in B. (D) Representative image of infected macrophages with white box indicating bacteria fully engulfed. Orthogonal xz and yz views highlight internalisation of rod bacterium. (E-F) THP-1 macrophage engulfment of UTI89 filaments at the single-cell level. X-axis shows engulfment with partial engulfment being < 0 and full engulfment > 0, as determined by nearest neighbour distance calculations. (E) LEX Filaments, (F) bladder model bacterial population. Scale bar = 20 μm. Data are the averages of 3 independent experiments with error bars representing the SEM. * indicates p < 0.05, ** p < 0.01 and ns indicates p > 0.05, determined by one-way ANOVA with multiple comparisons.

Bacterial populations (UTI89/pGI5 (msfGFP)) were isolated from the *in vitro* human bladder model and found to have a heterogeneous size distribution ranging from 0.7 μm– 235 μm, with ~ 63% classified as filamentous (bacterial cells > 10 μm in length) (Fig H in S1 Text). Despite many filamentous bacteria in this population, macrophage engulfment of the mixed population of bacterial cells was higher than expected in the gentamicin-protection assay; equivalent to a rod population grown in LB (Fig 6A), and 100-fold higher than a cephalexin-treated filament population grown in LB. Fluorescence microscopy was used to quantify the proportion of rods and filaments being internalised by macrophages. Importantly, as for LB-derived bacterial cells, bladder rods are internalised more frequently than bladder filaments (*p* = 0.004, Fig 6B). Further classification of engulfed bacterial cells as either partial (incomplete enveloping of bacteria by macrophage membrane) or full (bacteria completely enveloped by the macrophage membrane) engulfment revealed that overall, a significantly higher proportion of filaments were partially (rather than fully) engulfed than their rod counterparts from both bladder-and LB-derived environments. However, it appears that the environment from which the filaments are derived contributes to the ability of macrophages to attempt engulfment, as significantly more bladder-derived filaments were partially engulfed compared to LB-derived filaments (*p* = 0.003, Fig 6C and 6E). Furthermore, there was no significant increase in partial engulfment of bladder-derived rods compared to LB-derived rods (*p* > 0.05), suggesting that this environmental contribution to engulfment also shows a size-dependency. Thus, bacterial cell length remains an important factor influencing successful macrophage engulfment (internalization) even when filaments are derived from different environments. Additionally, our data indicates that specific environmental conditions may contribute to macrophage engulfment efficiency or dynamics, as macrophages attempt to engulf more filaments derived from a bladder infection model than those filaments grown in LB with cephalexin.

### UPEC filamentation in a mouse model and an *in vitro* human bladder model occurs independently of the SOS-induced filamentation genes, *sulA* and *ymfM*

One possibility to explain the increased attempts of macrophages to engulf human bladder cell-derived filaments, compared to filaments obtained in LB could relate to the mechanism by which filaments are formed in different environments. Previous *in vitro* studies have shown that cephalexin-induced filamentation in *E. coli* (strain SC1088), while inhibiting division by targeting PBP3, also induces the SOS response [60]. A major division inhibitor of the SOS response in *E. coli* is SulA, which is activated in response to DNA damage and interacts with FtsZ to inhibit the formation of Z rings and thus division [9,61]. It has been postulated that the SOS response, triggered by immune cell attack (oxidative damage), is required for UPEC filamentation and survival during infection in a mouse model of UTI [9,20,62]. However, UPEC transcriptomics and qPCR in an *in vitro* human bladder cell flow chamber model of infection revealed that the SOS response and *sulA* expression were not induced during UPEC filamentation, at least at the transcriptional level [16,18]. Furthermore, UPEC filamentation in both *in vitro* and *in vivo* models was found to be instead dependent on a mechanism involving a cell division regulator, DamX [16,45]. Filamentation of *E. coli in vitro* has also been observed to occur through multiple mechanisms in the absence of the SulA (Fig I in S1 Text) [16,63–65]. Given the apparent contradictions in conclusions from these previous studies, and to investigate potential differences in the physiology of filamentous bacteria produced by the cephalexin or UTI infection model conditions used here, we next sought to verify whether SOS division inhibition is the main mediator of UPEC filamentation observed during *in vivo* and *in vitro* UTI infection models.

**Fig 7:**
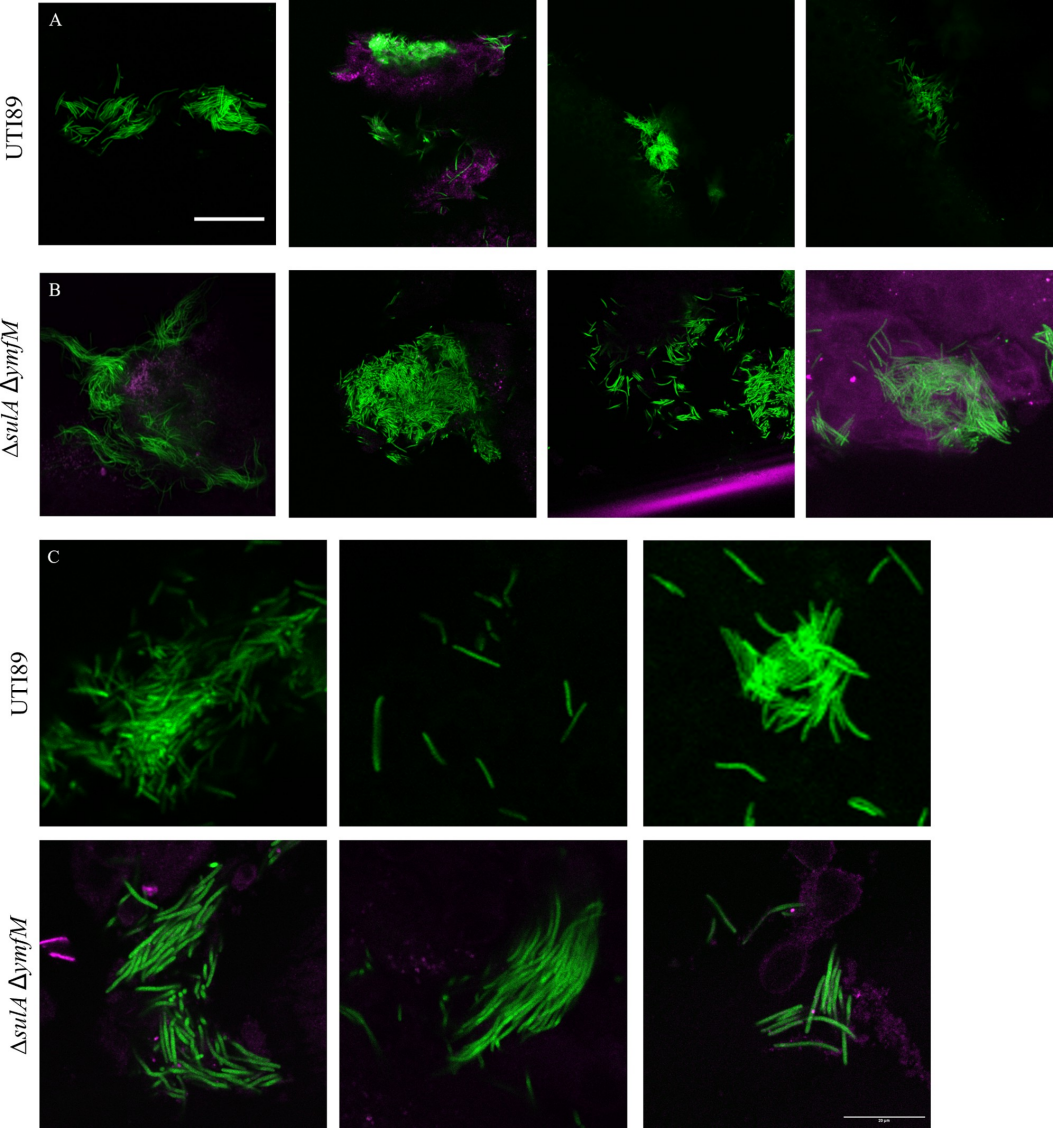
UTI89 mutants lacking both *sulA* and *ymfM* retain the ability to form filaments and replicate engulfment dynamics of wild-type UTI89. (A-B) Six hours post-infection, with (A) UTI89/pMAN01 (GFP) or (B) UTI89Δ*sulA*Δ*ymfM*/pMAN01 (GFP), C3H/HeN mouse bladders (magenta) were bisected and splayed on a silicone pad, fixed and imaged by an Olympus FV1000MPE microscope with a 20X objective (NA 0.75). Each bacterial strain was tested on a group of 4 mice with images representative from different mice. Scale bar = 50 μm. Representative images at higher magnification are shown for UTI89/pMAN01 (GFP) and UTI89Δ*sulA*Δ*ymfM*/pMAN01 (GFP) in (C). Scale bar = 20 μm.

We recently identified an e14 prophage gene, *ymfM*, that contributes to filamentation during the SOS response, and found that both *sulA* and *ymfM* must be removed to strongly suppress SOS filamentation [65]. We therefore investigated if UTI89 still undergoes filamentation in the absence of both *sulA* and *ymfM* in a mouse acute cystitis model. Mice were infected with UTI89Δ*sulA*/pMAN01 (GFP-expressing plasmid), UTI89Δ*ymfM/*pMAN01, UTI89Δ*sulA*Δ*ymfM*/pMAN01, or wild type bacterial cells, UTI89/pMAN01. Filaments, which were morphologically indistinguishable from the wild-type (Fig 7A and 7C), were observed by confocal laser scanning microscopy on the surface of four separately-infected mouse bladders each, in the absence of *sulA* or *ymfM* (Figs J and K in S1 Text), as well as in the double mutant (Fig 7B and 7C). The results clearly indicated that both these SOS-mediated filamentation genes are dispensable for UPEC filamentation in the mouse model of UTI. Consistent results were observed with the human bladder cell culture infection model, which enabled quantification of UPEC filament length (Fig H in S1 Text). Upon harvesting the bacteria during the filament-dispersal phase of infection, phase-contrast microscopy showed that UTI89Δ*sulA*Δ*ymfM*/pGI5 had high yields of highly filamentous bacteria present, indistinguishable from the wild-type UTI89/pGI5. This was complemented with flow cytometry, which showed similar distributions reflecting cell size for both strains, indicating that both strains contained the approximately same proportion of filamentous bacteria (Fig HC in S1 Text).

**Fig 8:**
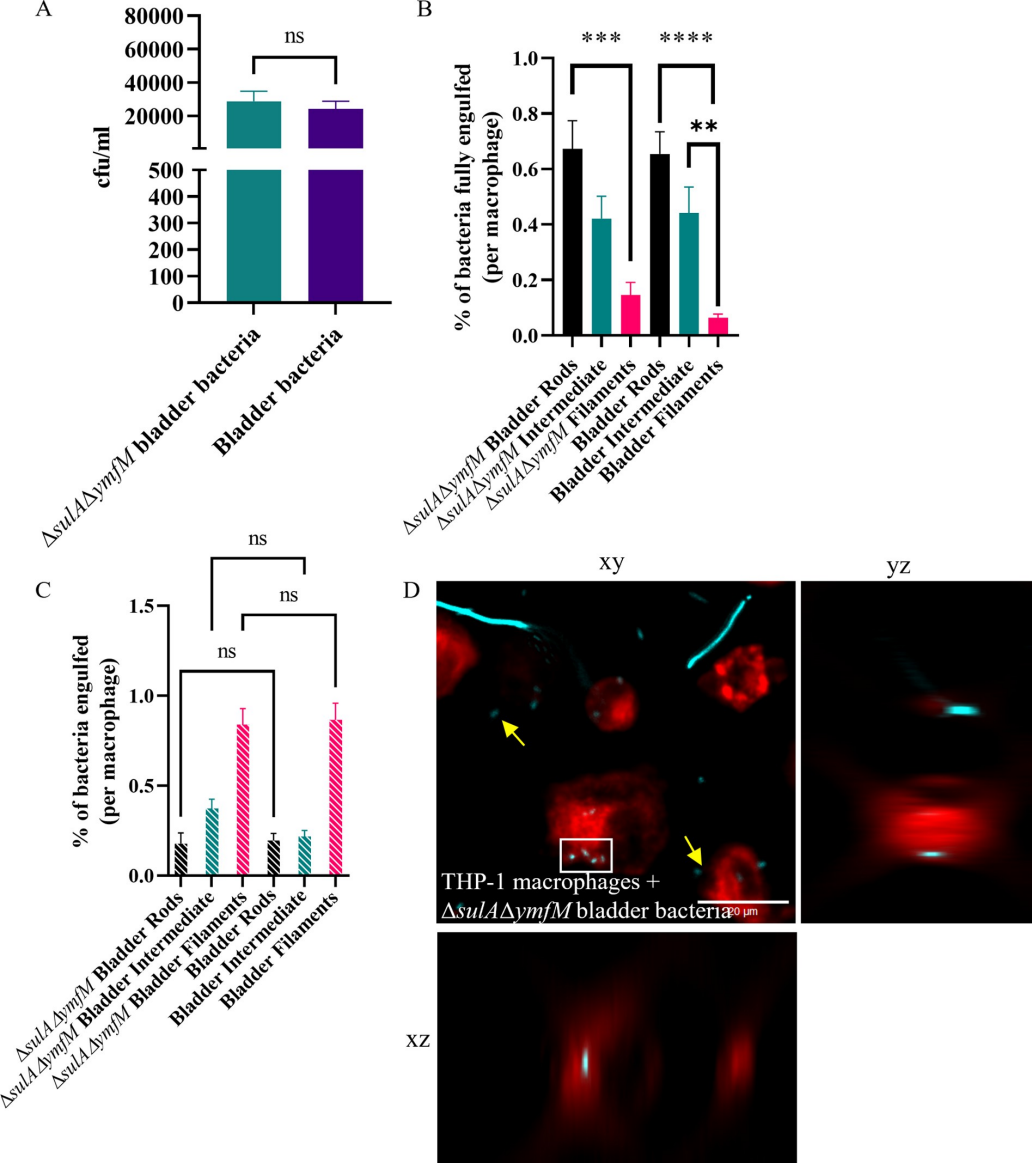
UTI89 mutants lacking sulA and ymfM retain the ability to form filaments and replicate engulfment dynamics of wild-type UTI89. (A-D) UTI89/pGI5 (msfGFP) or UTI89ΔsulAΔymfM/pGI5 (msfGFP) were isolated from an in vitro human bladder model (bladder bacteria or ΔsulAΔymfM bladder bacteria respectively). (A) THP-1 macrophages were infected (MOI 10) for 1 hour and bacterial loads were assessed 2-hours post infection in a gentamicin-protection assay. (B-D) Macrophages were infected with bladder bacteria or ΔsulAΔymfM bladder bacteria (cyan) at MOI 10, fixed after 60 minutes and stained with 1X CellMask Orange (red). Images were acquired using a DeltaVision Elite microscope with the 40X dry NA 0.60 objective. Internalisation of bacteria was determined using a FIJI macro on 3D image data. (B) Percentage of bacterial populations fully engulfed (number of bacteria fully engulfed/total number of bacteria counted) is normalised and presented as per macrophage to allow for comparisons despite variations in number of macrophages counted from microscopy images. (C) Percentage of bacterial populations partially engulfed per macrophage as determined by microscopy presented as in D. (D) Representative image of infected macrophages with white boxes indicating bacteria fully engulfed, and yellow arrows indicating extracellular bacteria. Orthogonal xz and yz views highlight internalisation of rod bacterium. Scale bar = 20 μm. Data are the averages of 3 independent experiments with error bars representing the SEM. * indicates p < 0.05, ** p < 0.01 and ns indicates p > 0.05, determined by one-way ANOVA with multiple comparisons.

To investigate the engulfment efficiency of filaments formed in the absence of *sulA* and *ymfM*, a population of heterogeneous lengths (UTI89Δ*sulA*Δ*ymfM*/pGI5 (msfGFP)) was obtained from the *in vitro* human bladder model. A gentamicin protection assay showed there was no difference in macrophage engulfment levels in the absence of both *sulA* and *ymfM* compared to UTI89/pGI5 (msfGFP) from the *in vitro* human bladder model (Fig 8A). Fluorescence microscopy indicated that macrophage engulfment dynamics of both rods and filaments lacking both *sulA* and *ymfM* were comparable to their wild-type counterpart: there was still a preference (2.4-fold, *p* = 0.018) for internalisation of rods compared to filaments (Fig 8B and 8D), and macrophages partially engulfed Δ*sulA*Δ*ymfM* filaments grown in the *in vitro* bladder (0.56%) in similar proportions to UTI89 bladder filaments from the same model (0.64%) (Fig 8C). Together, these data show that UTI89 is still able to filament during *in vivo* and *in vitro* infection models in the complete absence of the two known SOS filamentation genes *ymfM* and *sulA*, and that macrophages interact with these filaments in a similar way to wild-type human bladder-derived filaments. These findings also indicate that the cephalexin-induced filaments, which are expected to result from both a direct cephalexin-induced inhibition of division and through SOS-induction, are physiologically distinct from the *damX*-dependent (SOS independent) filamentation observed in our *in vivo* and *in vitro* UTI infection models. Physiological differences associated with these different filament types appear to underpin the preferential macrophage targeting of the infection-derived filaments.

## Discussion

Bacterial filaments have historically been regarded as by-products of growth in ‘stressful’ or toxic environments. However, we are now beginning to understand that morphological plasticity may provide an advantage to many bacteria in a variety of environments. One such example is that UPEC filaments delay/inhibit engulfment by immune cells such as neutrophils and macrophages in a mouse model of UTI [8,20]. Whilst it is assumed that this reduced engulfment is due to increased bacterial length, the exact advantage of filamentation has remained unclear. There may also be an assumption that all filaments are biologically, chemically, and structurally the same, and filament length or the environmental cues to induce filamentation are often overlooked as factors that may influence macrophage engulfment. We have begun to address these fundamental questions by examining the minimum bacterial cell length needed to significantly reduce engulfment by macrophages, and by systematically investigating biophysical parameters such as size and shape, and physiological parameters such as surface and environment, to understand what factors contribute to the filament survival advantage in the presence of human macrophages.

During infections bacteria are not inert or dead. Here we used live, viable populations of bacteria, more truly reflective of how they interact with immune cells. For the first time, to the best of our knowledge, this study identified conditions that produced viable, metabolically active and transient filaments of known lengths. This is critical to understanding how a changing morphology provides a survival advantage in the environment. In support of previous studies [8,20,21], we found that UPEC filaments (bacteria > 10 μm long) were engulfed significantly less than rods–up to 150-fold less ‐ (Fig 1B–1D) and in mixed populations rods were engulfed preferentially over filaments (Figs 2, 6 and 8). Further analysis found that UPEC populations, exposed to antibiotics, only needed as little as a doubling in average bacterial cell length to be less effectively engulfed, and the level of engulfment decreased exponentially as bacterial cell length increased (Fig 3). In simple terms, getting as long as possible in the environment could provide bacteria with an increased survival advantage. However, the energetic burden of switching from bacterial cell division to filament formation and back is unknown. Previous studies have shown links between bacterial central metabolism, cell division and cell size [66,67], and proteomics analysis of *E. coli* filaments (induced by ampicillin treatment of persister cells) has demonstrated changes in carbon metabolism [68], but the direct link between filamentation and metabolism has not yet been fully investigated. Regardless of the energetic cost or benefit of filamentation, it is clear that bacterial cell length is a major contributor to macrophage engulfment efficiency.

Computational predictions of engulfment [25] and practical studies using inert particles [50] or bacterial samples [21,22] have all demonstrated that successful engulfment of filamentous shapes, regardless of their size, relies on the phagocyte attaching to the pole of the target. In fact, the actual engulfment of *Legionella* filaments occurs at the same speed as rods but is delayed by the macrophage trying to reorient the filament to find the pole [21]. The bacterial populations from the *in vitro* bladder model used here included bacterial cells of various sizes with some filaments being over 250 μm long, over 10-fold longer than cephalexin-induced filaments. Investigating engulfment efficiency (comparing partially internalised and fully internalised bacteria) in this study revealed that macrophages partially internalised more filaments compared to rods, in both LB-derived and bladder-derived populations, with increased attempted engulfment of filaments from the *in vitro* bladder model. While not captured during the time frame of our assay, we hypothesize that this captured partial internalisation may eventually result in greater numbers of fully internalised filaments with engulfment being delayed by the requirement of finding the pole of the very long filaments, such as those in the bladder-derived population. A previous theoretical (computational) study showed that increasing sphere size would decrease engulfment by increasing the time required for membrane wrapping of the target [69]. Our work supports this hypothesis, we observed that as sphere volume increases (‘big spheres’), they are engulfed 67-fold less than the smaller spheres. Therefore, for UTI89 of different shapes, bacterial cell size remains an influence on effective engulfment.

While the size and shape of engulfment targets are closely linked, the shape of targets can additionally be described by aspect-ratio, curvature and circularity. On examining how UTI89 cell shape might affect macrophage engulfment, we found that spherical cells are preferentially engulfed over rods, even when rods have a smaller volume. Spherical bacterial cells are also engulfed significantly more than filaments of approximately equal volume, indicating that UTI89 shape, as well as length and size, influence the ability of macrophages to engulf these bacteria (Fig 4A). This result builds on previous engulfment studies, using viable bacteria instead of inert particles. These previous studies found that engulfment relies on the circularity of nanoparticles [70], and that macrophage engulfment of spherical polystyrene particles is greater than that of high-aspect ratio shapes such as “worm-like” particles, of equal volumes [50]. Modes of entry of targets through a membrane have been predicted to differ based on the size, aspect ratio and curvature of particles, although the predicted mode of entry for high-aspect ratio and round tipped particles was noted to be different to that demonstrated practically for bacterial filaments [25]. Thus, our results both support and build on those described in previous studies and demonstrate an interplay between size and shape of bacteria that affects macrophage engulfment.

Whilst spheres produced by mecillinam treatment were viable in this study, it was noted that viability was reduced for mecillinam/cephalexin treated spheres compared to untreated rods. This could be due to a number of reasons: cephalexin and mecillinam are known to have a synergistic effect on *E. coli* viability [71], with links to the bacterial metabolite bulgecin [72] as well as other metabolic changes induced by mecillinam alone [73]. Treatment of *E. coli* with sub-lethal mecillinam has also been demonstrated to induce bacterial cell wall changes, making it more isotropic and having altered chirality [74]. Although the chemical composition of the cell wall has been reported to be unaltered after mecillinam treatment [74], the addition of cephalexin used in this study may contribute further to cell wall changes. It has been reported that cephalexin increases the amount of structural change in the bacterial cell wall when used in addition to another penicillin antibiotic, ampicillin [75]. So, while increasing the size of spheres did reduce engulfment, further investigation on the role of surface composition should be performed to determine whether the macrophage engulfment changes were due to alterations in cell wall composition. Genetic manipulation of *E. coli* through deletion of the rod-shape determining protein MreB [76] could be informative in investigating macrophage engulfment of round bacterial cells in the absence of antibiotic treatments.

The role of Type 1 fimbriae and the adhesin FimH in both establishing infection and engulfment through binding to mannosylated glycoproteins on mammalian cells has been well established for rod-shaped *E. coli* [77]. A previous study deleted the entire *fim* operon from a K-12 strain of *E. coli* and found that these bacteria do not get engulfed by RAW 264.7 mouse macrophages [78], supporting the theory that fimbriae are essential for phagocytosis of *E. coli*. We found indirect support for the importance of fimbriae in engulfment by blocking the ability of UTI89 to bind to mannose and observing a reduction in engulfment by macrophages (Fig 5). The equal drop in engulfment of rods and filaments indicates that there is no difference in their ability to interact with the mannosylated host cell surface, and that size/shape of bacteria is the primary driver of engulfment efficiency and not an inability of filaments to bind to the mannosylated surface of macrophages. However, low-level engulfment of both rods and filaments was still observed even after the blocking of available mannose-binding sites, suggesting that binding to the mannosylated host cell surface may not be the only pathway for engulfment. Macrophages have numerous receptors on their cell surface, performing a variety of functions that contribute to phagocytosis: some initiating signalling pathways to induce phagocytosis directly while others work in conjunction with these phagocytic receptors to detect molecular patterns of pathogenic invaders [79]. The vast numbers and types of receptors capable of phagocytosing different targets through various pathways [79] coupled with the range of glycans and glycolipids on the UPEC cell surface [56,80], and the ability of UPEC to switch expression of its surface structures on/off [81,82], supports this hypothesis that alternate engulfment pathways do exist.

Macrophage engulfment of bacterial populations isolated from an *in vitro* bladder model show that they attempt to engulf more filaments compared to filaments grown in defined nutrient medium. This environmental effect during filament development in the *in vitro* bladder model is currently unknown, however could be due to complement-coating or opsonization which have previously been shown to enhance the engulfment of certain pathogens [83,84]. It has also been demonstrated that UPEC undergoes filamentation when exposed to urine in both the presence and absence of host bladder cells [18,28], which raises the possibility that unknown component(s) of urine could influence macrophage engulfment. UTI89 planktonic bacteria cultured in human urine can be devoid of type 1 fimbriae, and switch to expressing type 1 fimbriae when in sessile populations [85]; further investigation of the role in type 1 fimbriae during engulfment would address this.

We found that UTI89 was still able to filament when exposed to urine in the complete absence of the two known SOS filamentation genes *ymfM* and *sulA* in both an *in vivo* mouse UTI model and an *in vitro* human bladder cell model. A control showed this double mutant cannot form filaments on SOS induction in culture, as expected [65]. Previously, deletion of *sulA* was reported to abolish filamentation in an *in vivo* mouse model of UTI [20], although an *in vitro* flow cell model of UTI and transcriptomics showed no SOS or *sulA* induction during filamentation [16,18]. Furthermore, UPEC filamentation in both *in vitro* and *in vivo* models of acute UTI has been shown to be dependent on an alternative pathway requiring *damX* [16,45]; no filaments were observed in UTI89Δ*damX* in either infection model, in the presence of *sulA*. The reasons for the apparent discrepancy in results on the role of *sulA* in UPEC infection-mediated filamentation are currently unknown, though we are concerned that the use of P1 transduction to make the previous UTI89. Δ*sulA* strains [20,62], which can co-transfer up to 100 kb of DNA from the non-pathogenic K-12 mutant donor strain [86], might have attenuated virulence independently of *sulA*. Our results, combined with those previously reported on the strict requirement for *damX* in infection-related filamentation (IRF), support the proposal that known components of the SOS response (*sulA* and *ymfM*) are dispensable for UPEC filamentation during the establishment of UTIs in the *in vitro* flow human bladder cell model of UTI and mouse acute cystitis models of UTI.

We also demonstrated that the increased ability of macrophages to partially engulf human bladder-derived filaments is unchanged in the absence of *ymfM* and *sulA*. Our data are consistent with a previous study where both wild-type and Δ*sulA* UTI89 filaments (induced by mitomycin C treatment) were readily engulfed by human and mouse polymorphonuclear leukocytes [20]. Future studies observing engulfment of UTI80 Δ*sulA*Δ*ymfM* filaments *in vivo* would further confirm our observations. Overall, there are likely many unknown factors that induce filamentation under different environmental conditions, and how these influence the dynamics and efficiency of macrophage engulfment requires future investigations.

In conclusion, the data presented here contributes to our understanding of why bacteria filament during UTIs but also highlights the pitfalls of making general conclusions when discussing bacterial filamentation. There are many factors that influence the interactions between the bacteria and host, and it is becoming increasingly clear that these interactions are incredibly complex and warrant further study. Some of the bacterial factors that affect host macrophage engulfment (and thereby infection responses) were investigated here, revealing that for *E. coli* UTI89, size, shape, surface and growth environment can cause variations in the engulfment effectiveness by human THP-1 macrophages. Furthermore, the long-known SOS response of bacteria still has many unknown aspects and the novel observation of engulfment differences of filaments formed by antibiotics in LB and within bladder epithelial cells in urine open new research directions for future exploration. Improving our knowledge of bacterial filaments will allow a deeper understanding of the process of infection, how pathogens interact with our immune response, and ultimately uncover novel avenues for non-antibiotic treatments which are urgently needed to treat resistant pathogens.

## Methods

### Ethics statement

The mice experiments were conducted according to the national guidelines by the National Danish Animal Care Committee. The study was approved by the Danish Animal Experimentation Inspectorate under the Ministry of Food, Agriculture and Fisheries, license number 2015-15-0201-00480. Human urine collection was approved by the University of Technology Sydney Human Research Ethics Committee (HREC no. 2014000452). Written consent was obtained from participants for use of their sample for research purposes.

### Bacterial strains and plasmids

Table C in S1 Text contain lists of bacterial strains and plasmids.

### Conditions to establish filamentation

#### Growth of rods and antibiotic-induced filamentous bacteria

All bacterial strains were grown statically at 37°C unless otherwise stated. Twenty-five ml of LB was inoculated from an overnight culture to a starting OD_600_ = 0.05 and incubated statically until early exponential phase (OD_600_ ~ 0.2). One culture then acted as a control with no antibiotic addition, whilst the remaining two had 2.5 μg/ml (LEX rods) and 10 μg/ml (LEX filaments) of cephalexin added. Cultures were grown until OD_600_ of control reached 0.7–0.8, before bacterial cells were centrifuged at 3500 *g* for 5 minutes and resuspended in PBS to CFU/ml of 2x10^7^. UTI89/pGI5 (msfGFP), UTI89Δ*fimH* and UTI89Δ*fimH*/pGI5 (msfGFP) was grown the same way with the addition of 100 μg/ml spectinomycin for selection of the pGI5 (msfGFP) plasmid in LB.

For mixed populations rods and LEX filaments were grown as described above with the following addition. After final centrifugation cultures were resuspended in PBS to a final CFU/ml of approximately 2x10^7^ of which 24% were rods (approximately 0.53 x 10^7^ CFU/ml) and 76% were filaments (approximately 1.6 x 10^7^ CFU/ml).

For ciprofloxacin-induced filaments UTI89 was grown the same way as described for cephalexin treatment with the following additions. After growth to early exponential phase one culture then acted as a control with no antibiotic addition, whilst the remaining two had 3.75 ng/ml (CIP rods) and 15 ng/ml of ciprofloxacin (CIP filaments) added. Cultures were grown until OD_600_ of rods reached 0.7–0.8, before centrifuged at 3500 *g* for 5 minutes and diluted to an OD_600_ of 0.1 in fresh LB and grown for 1.5 hours in the presence of the same concentrations of ciprofloxacin. Cultures were centrifuged at 3500 *g* for 5 minutes and resuspended in PBS to CFU/ml of 2x10^7^.

#### Expression of *ftsZ-yfp*

First, 5 ml of LB with ampicillin (100 μg/ml) and 0.2% (v/v) glucose solution (to repress expression of *ftsZ-yfp* from pLau80) was inoculated with a single colony of UTI89/pLau80 or UTI89/pG15/pLau80 and allowed to grow overnight at 37°C. The overnight culture was diluted in 20 ml LB with ampicillin (100 μg/ml), spectinomycin (100 μg/ml) and 0.2% (v/v) glucose solution so that the OD_600_ = 0.05. Cultures were grown at 37°C for 2 hours before being centrifuged at 3500 *g* and washed twice with fresh LB media to remove glucose. Cultures were diluted to OD_600_ = 0.1 in 20 ml LB with ampicillin (100 μg/ml), spectinomycin (100 μg/ml) and either 0.2% (v/v) glucose solution (repression of pLau80) or 0.2% (v/v) arabinose solution (expression of pLau80). Cultures were grown at 37°C for 2 hours and then cultures were centrifuged at 3500 *g* for 5 minutes and resuspended in PBS to CFU/ml of 2x10^7^.

#### Growing filaments of different lengths

Cultures were grown as previously described for antibiotic induced filaments and expression of *ftsZ* with the following additions. Cultures were grown to different ODs before induction of filamentation through addition of cephalexin or 0.2% (v/v) arabinose. They were collected at the same end point, an OD_600_ equivalent to an untreated culture (0.7–0.8). Cultures ended up being grown at 37°C in the presence of 10 μg/ml of cephalexin for 1 hour 30 min, 1 hour 15 min, 1 hour, and 45 min, or in the presence of 0.2% (v/v) arabinose solution for 2 hours 15 min, 1 hour and 30 min. Cultures were centrifuged at 3500 *g* for 5 min and resuspended in PBS to CFU/ml of 2x10^7^.

#### Growing bacteria in different shapes

Cultures were grown as previously described for antibiotic induced filaments with the following additions. Cultures were grown at 37°C for 1 hour 15 min to an OD_600_ ~ 0.2 (early exponential phase). Cultures had 10 μg/ml mecillinam only or 10 μg/ml mecillinam and 10 μg/ml cephalexin added and were grown at 37°C for 1 hour 30 min before they were centrifuged at 3500 *g* for 5 minutes and diluted to an OD_600_ of 0.1 and grown for 1 hour 30 min in the presence of the same concentrations of antibiotics. Cultures were centrifuged at 3500 *g* for 5 min and resuspended in equal volumes of PBS.

#### Quantification of length and volume

Bacteria were fixed with 3.7% formaldehyde (v/v) for 1 hour at room temperature prior to measurement. Lengths of bacteria were measured through phase contrast microscopy on the Zeis Axioplan2 with accompanying software. Volumes of bacteria were measured by the Multisizer M4e Coulter Counter Analyzer (Beckman). Samples fixed in formaldehyde solution were diluted to 1% (v/v) in filtered IsoFlow Sheath Fluid (Beckman). Of this, 200 μl was run through a 50 μm aperture tube, and data were collected over 400 bins ranging from 0.5 μm^3^ to 100 μm^3^ were measured.

### Growth and culture of THP-1 monocytes

The human monocytic cell line used was THP-1 (ATCC TIB-202), which was derived from an acute monocytic leukaemia patient. The THP-1 cells were cultured at 37°C, 5% CO_2_ in RPMI-1640 medium containing 10% (v/v) FBS (fetal bovine serum, heat inactivated) and 1% (v/v) GlutaMAX. Approximately 2x10^5^ THP-1 cells/ml were added to a 24 well plate (1ml per well) and were stimulated to form macrophages with 30 ng/ml of PMA (Phorbol 12-myristate 13-acetate) for 48 hours. THP-1 cells were washed and resuspended in fresh RPMI-1640 medium containing 10% (v/v) FBS and 1% (v/v) GlutaMAX (RPMI culture media) and incubated at 37°C, 5% CO_2_ for 24 hours before infection with bacteria.

### Gentamicin-protection assay

The bacterial cultures were added to macrophages at an MOI of 10 (MOI 10) per well (approximately 2x10^6^ viable bacteria per well determined by CFU/ml). Plates were centrifuged at 1000 *g* for 5 minutes and incubated for 1 hour at 37°C and 5% CO_2_. THP-1 and bacterial cells were then treated with 200 μg/ml gentamicin for 1 hour at 37°C and 5% CO_2_. Cells were washed twice with PBS and lysed with 0.1% (v/v) Triton X-100 for 15 min, serially diluted in PBS, plated on LB agar and incubated at 37°C for 16–18 hours. Colonies were then counted, and CFU/ml was calculated. This was done in technical triplicate, with at least 3 biological replicates.

For blocking with methyl α-D-mannopyranoside the gentamicin-protection assay was performed as described above with the following additions. Before the addition of bacteria, RPMI culture media was supplemented with 3% (w/v) methyl α-D-mannopyranoside (α-D-MP) [58]. The bacterial cultures (also in 3% (w/v) α-D-MP) were added to macrophages at an MOI 10 per well (approximately 2x10^6^ bacteria per well).

For microscopy analysis macrophages were stimulated the same way and were infected with the MOI 10 for 1 hour. However, CellCarrier-96 well Black plates were used for the assay with 1x10^5^ THP-1 cells/ml added (200 μl per well). After the initial 1-hour incubation at 37°C in 5% CO_2_ macrophages were stained with a 1X CellMask Orange Plasma membrane Stain at 37°C for 10 minutes in the dark, washed twice with PBS and fixed by adding 3.7% (v/v) formaldehyde at 37°C for 20 minutes in the dark.

### Imaging

Fixed and live bacteria were viewed using phase contrast and widefield fluorescence microscopy with a Zeiss Axioplan 2 fluorescence microscope and a 100X oil immersion NA 1.4 Plan Apochromat objective lens. The light source was a 100 W high pressure mercury lamp that passed through filter blocks for observing GFP (Filter set 09; 450–490 nm BP excitation, 515 nm LP barrier filter) and propidium iodide (Filter set 15; 546/12 nm BP excitation, 590 nm LP barrier filter). Images were taken using a Zeiss AxioCam MRm camera and analysed using AxioVision software version 4.8 (Zeiss). For widefield fluorescence microscopy of UTI89/pGI5 (msfGFP), the exposure time was maintained at 200 ms. For imaging membrane integrity staining of GFP and propidium iodide the exposure times were maintained at 100 ms and 200 ms respectively.

Three-dimensional z-stack acquisition was performed using a DeltaVision Elite widefield fluorescence microscope (GE Healthcare) using a 40X objective (OLYMPUS LUCPLANFLN 40X dry objective NA 0.60). The filter sets used were GFP (464–492 nm excitation) and TRITC (531–565 nm excitation). Each stack acquired consisted of 25–30 optical slices with intervals of 1.45 μm (this was optimised for subsequent deconvolution microscopy). Stacks were deconvolved using the softWoRx Enhanced Ratio deconvolution method (GE Healthcare).

### Digital analysis of microscopy images of engulfment

Analysis of deconvolved images was done using FIJI software utilising both available plugins and custom macros. Thresholding and masking to identify the macrophages was based on membrane staining with 1X CellMask Orange. Bacteria were identified by msfGFP expression from the pGI5 plasmid. Nearest neighbour distance calculations were used to identify how close bacteria were to macrophages including bacteria within the membrane of a macrophage. For rods, a fully internalised bacterium was defined as having a value of > 1.0. For filaments, a fully internalised filament was assigned two values for the closest and furthest aspect of the bacteria and if both were negative this correlated to a fully internalised filament, while if only one was negative this indicated partial engulfment. The total number of macrophages in an image stack were counted, and the total bacteria per image stack to enable analysis and quantification of population percentages. A minimum of 4 images were analysed per time point. A minimum of 2 biological replicates were performed. Data were measured per image, with an average calculated from the replicate images. Images were not taken by a blinded investigator, but the software defined the measurements.

### *In vitro* bladder infection model

The set-up was based on a previously established approach [18,44,45], with minor modifications. Briefly, on day one, flow chambers (IBIDI μ-Slides I^0.2^ Luer, Cat#: 80166) were seeded with PD07i epithelial bladder cells at a concentration of ~ 3x10^6^ cells in EpiLife Medium (Gibco, #MEPI500CA) supplemented with growth supplements and antibiotics (HKGS, #S0015, and 100μg ml^-1^ Pen/Strep, SIGMA #P4333). Seeded channels were left overnight to allow bladder cells to adhere and multiply into a confluent layer. The next day, flow channels were connected to New Era pumps via tubing and 20 ml disposable syringes. Flow (15 μl min^-1^) of fresh EpiLife (supplemented with HKGS) without antibiotics was maintained for 18–20 hours. On day three, to induce infection, bladder cells were exposed to bacterial cultures (i.e., UTI89 grown statically at 37°C overnight) at a concentration of OD_600_ 0.2 for 15 min at a flow rate of 15 μl min^-1^. Following this step, the media was changed back to EpiLife (supplemented with HKGS), after an initial flow of 100 μl min^-1^ to flush out the excess bacteria, and flowed for an additional 9 hours to allow bacteria to adhere to and invade the epithelial bladder cells. This step was followed by flow (15 μl min^-1^) of EpiLife (supplemented with HKGS) in the presence of 100μg/ml gentamycin for 20 hours to allow for the formation of intracellular bacterial communities (IBCs), as well as to remove any lingering extracellular bacteria. Following these 20 hours, the media was changed to sterile filtered human urine (with pH between 5.12–5.83 and Urine Specific Gravity of at least 1.024 g mL^-1^, [44]) with flow (15 μl min^-1^) for 18–20 hours to induce filamentation and dispersal of the bacteria from the bladder cells. Filaments were collected from the back-opening of the flow channels.

### Mouse cystitis model

The model is based on Hung, Dodson [87]). Deletion strains were constructed in UTI89 by the well-established lambda Red recombinase method [88]. A total of 4 strains were constructed ‐ UTI89/pMAN01, UTI89Δ*sulA*/pMAN01, UTI89Δ*ymfM*/pMAN01, UTI89Δ*sulA*Δ*ymfM*/pMAN01. After 1 week of acclimatization, 8-9-week-old female C3H/HeN mice (Janvier Labs, France) were anaesthetized followed by inoculated by transurethral catheterization with approx. 1–2 x 10^7^ cfu of either strain diluted in 50 μl PBS (as determined by OD_600_). After 6 hours of infection, the mice were euthanized, and bladders were collected and bisected. Each sample was then splayed on a silicone pad and fixed in 3% (v/v) paraformaldehyde followed by confocal laser scanning microscopy using an Olympus FV1000MPE microscope with a UPLSAPO 20X/0.75NA objective and Olympus FV10-ASW software. Each strain was tested on a group of 4 mice.

### Statistical analysis

*P*-values were determined by one-way ANOVA with multiple comparisons (for 3 or more conditions), or t-test (for less than 3 conditions) through GraphPad Prism software. Tukey, Šidák or Welch’s tests were used for multiple comparisons, and Tukey or Welch’s test for t-tests on less than 3 conditions, depending on the test for equal variance. Power analysis was performed on raw data to confirm the sample size required (α = 0.05 and desired power = 0.8). Following this, data were normalised if required. Datasets used in analysis for this study are available in Dryad [89].

## Supporting information

S1 Text **Fig A. Phase contrast images of rods and filaments induced by cephalexin, ciprofloxacin and ftsZ-yfp expression**. (A-D) UTI89 cells were untreated (rods) or treated with 10 μg/ml cephalexin (LEX filaments) or 15 ng/ml ciprofloxacin (CIP filaments). (E-F) UTI89/pLau80 cells were treated with 0.2% (v/v) glucose (FtsZ rods) or 0.2% (v/v) arabinose (FtsZ filaments). Samples were collected and fixed with 3.7% (v/v) formaldehyde. (A-F) Phase contrast images were acquired and cell lengths measured using a Zeiss Axioplan 2 microscope with the 100X oil immersion NA 1.4 objective. Representative phase contrast images of (A) UTI89 rods without cephalexin, (B) UTI89 LEX filaments, (C) UTI89 rods without ciprofloxacin, (D) UTI89 CIP filaments, (E) FtsZ rods, and (F) FtsZ filaments. White text in bottom left corner of images indicates average lengths of the populations. Data are from 2 independent experiments with n = 110–347. Scale bar = 5 μm. **Fig B. Membrane permeability of rods and filaments induced by cephalexin, ciprofloxacin and ftsZ-yfp expression.** UTI89 was treated with either cephalexin (rods: untreated, LEX rods: 2.5 μg/ml, LEX filaments: 10 μg/ml) or ciprofloxacin (rods: untreated, CIP rods: 3.75 ng/ml, CIP filaments: 15 ng/ml). UTI89/pLau80 was treated with 0.2% glucose (FtsZ rods) or 0.2% arabinose to induce expression of ftsZ-yfp (FtsZ filaments). Cells were stained with SYTO 9 and propidium iodide from LIVE/DEAD BacLight Bacterial Viability Kit, and phase contrast and fluorescence images were acquired and analysed using a Zeiss Axioplan 2 microscope with the 100X oil immersion NA 1.4 objective. (A-C) Percentage of the bacterial populations with non-permeable membranes as quantified by microscopy. Data are averages of 2 independent experiments with error bars representing the SEM, n = 131–191. **Fig C. Metabolic viability of rods and filaments induced by cephalexin, ciprofloxacin and ftsZ-yfp expression.** UTI89 was treated with either cephalexin (rods: untreated, LEX rods: 2.5 μg/ml, LEX filaments: 10 μg/ml) or ciprofloxacin (rods: untreated, CIP rods: 3.75 ng/ml, CIP filaments: 15 ng/ml). UTI89/pLau80 was treated with 0.2% glucose (FtsZ rods) or 0.2% arabinose to induce expression of ftsZ-yfp (FtsZ filaments). Cells were stained with BacTiter-Glo™ Reagent from BacTiter-Glo Microbial Cell Viability Assay. ATP luminescence was quantified by the Tecan plate reader M200. (A-C) The relative luminescence was calculated as a percentage of the control ‘Rod’ populations (A-B: untreated rods, C: FtsZ rods) with the dashed line representing these populations at 100%. Data are the averages of 2 independent experiments with error bars representing the SEM. **Fig D. Reversion of UTI89 filamentous populations occurs on removal of cephalexin, ciprofloxacin or FtsZ-YFP inducer.** After induction of filamentation with 10 ug/ml cephalexin, 15 ng/ml ciprofloxacin or 0.2% (v/v) arabinose for FtsZ-YFP expression, cultures were centrifuged and diluted into fresh LB and incubated at 37°C. Fixed samples were taken for cell length measurement using phase contrast microscopy with Zeiss Axioplan2 using the 100X oil immersion NA 1.4 objective. (A-C) Cell length distributions over time following the removal of (A) cephalexin, (B) ciprofloxacin, or (C) 0.2% (v/v) arabinose. Data are from 2 independent experiments, n = 102–355. (D) Phase contrast images showing reversion of filaments to rods over time. T indicates hours after cephalexin removal. Images are representative from 1 experiment. Scale bar = 5 μm. **Fig E. UTI89/pGI5 (msfGFP) LEX filaments do not revert significantly to rods during a 1-hour incubation in RPMI culture media.** UTI89/pGI5 (msfGFP) cells were treated with 10 μg/ml cephalexin (LEX filaments; before redilution in RPMI). To replicate gentmaicin-protection assay conditions cultures were rediluted in RPMI culture media without cephalexin for 1 hour at 37°C 5% CO_2_ (LEX filaments in RPMI). Samples were collected and fixed with 3.7% (v/v) formaldehyde. Phase contrast images were acquired and cell lengths measured using a Zeiss Axioplan 2 microscope with the 100X oil immersion NA 1.4 objective. Scatterplot of UTI89/pGI5 (msfGFP) cell length distributions are from 2 independent experiments with n = 109–113. Horizontal lines indicate mean. **Fig F. The relationship between cell concentration (cfu/mL) and biomass (OD**_**600 nm**_**) in cultures containing rods and filamentous cells.** UTI89 cultures were either untreated (rods), or treated with 2.5 μg/ml cephalexin (LEX rods) or with 10 μg/ml cephalexin (LEX filaments). Samples were collected over time for OD_600_ and cfu/ml measurements. The yellow dashed line indicates the time of addition of cephalexin in LEX rods and LEX filaments cultures), and the blue dashed line indicates the time when cells were collected for determining cfu/mL and for length/volume/viability analysis and gentamicin protection assays in this study. (A) The biomass of the three populations over the growth and treatment period. (B) The number of cfu/mL in the relative to the biomass of the populations (absorbance). Data are from 2 independent experiments, with graphs representative of 1 independent experiment. After addition of 10 μg/ml cephalexin (LEX filaments), the biomass (cell growth) continues to increase, but the number of cfu/mL does not, showing that cell division but not growth has been arrested in these cultures to form the filaments observed. This indicates that as rods elongate into filaments they remain viable over time; one filament results in one cfu on average. **Fig G. UTI89 containing pGI5 showed no difference in macrophage engulfment in either rod or LEX filament populations.** UTI89 cells were grown as separate rod or LEX filament populations before combining to produce a heterogeneous population. One population contained pGI5 (rods; black bars or filaments; red bars) to allow selective antibiotic plating. There was no significant difference in a gentamicin protection assay if wither rod or filament populations contained the plasmid as either a homogenous (rod or filament) or heterogenous (mixed) population. Data are the averages of 4 independent experiments (A and B) or 2 independent experiments (C) with error bars representing the SEM. Statistical significance determined by one-way ANOVA with multiple comparisons or Welch’s t-test. **Fig H. Filamentation of bacteria isolated from the in vitro human bladder model.** (A) UTI89/pGI5 (msfGFP) were grown in and isolated from an in vitro human bladder model (bladder bacteria). Samples were collected and fixed with 3.7% (v/v) formaldehyde. Phase contrast images were acquired and cell lengths measured using a Zeiss Axioplan 2 microscope with the 100X oil immersion NA 1.4 objective. Cell lengths of bladder bacteria ranged from 0.72 μm to 253 μm long. Data are from 2 independent experiments with n = 337. Horizontal line indicates mean. (B) Representative phase contrast microscopy of UTI89/pGI5, ΔymfM and ΔsulAΔymfM mutants isolated from an in vitro human bladder model, showing filamentous cells for each strain. Scale bar = 5 μm (C). Flow cytometry of UTI89/pGI5 (red), ΔymfM/pGI5 (magenta) and ΔsulAΔymfM/pGI5 (green) mutants showing similar peaks indicating a bacterial population with similar morphology. The y-axis was normalised to mode to clarify the different peak positions; x-axis depicted the side scatter measurement (SSC-A) which corresponds to length of the bacteria. **Fig I. Filamentation of UTI89 in the presence of mitomycin C.** UTI89 and single sulA or ymfM mutants, and the double sulA ymfM mutant, were grown statically in LB to mid-log phase. 300 ng/ml mitomycin C was added and cells were incubated for a further 1h. Samples were observed by phase contrast microscopy (A) using a DeltaVision Elite inverted microscope with a 100x NA 1.4 objective, and average cell lengths were measured using FIJI (B). Results are from two independent experiments. Scale bar = 10μm. **Fig J. UTI89 mutants lacking sulA and ymfM retain the ability to form filaments.** (A-B) Six hours post-infection, with (A) UTI89ΔymfM /pMAN01 (GFP) or (B) UTI89ΔsulA/pMAN01 (GFP), C3H/HeN mouse bladders (magenta) were bisected and splayed on a silicone pad, fixed and imaged by an Olympus FV1000MPE microscope with a 20X objective (NA 0.75). Each bacterial strain was tested on a group of 4 mice with images representative from 1 mouse. Scale bar = 50 μm. **Fig K. Filamentation of bacteria isolated from a mouse cystitis model.** Six hours post-infection, with UTI89ΔymfM /pMAN01 (GFP), UTI89ΔsulA/pMAN01 (GFP) or UTI89 ΔsulA ΔymfM /pMAN01 (GFP), C3H/HeN mouse bladders (magenta) were bisected and splayed on a silicone pad, fixed and imaged by an Olympus FV1000MPE microscope with a 20X objective (NA 0.75). Semi-quantification of degree of filamentation was assessed for 3 isolated bladders (2 for ΔymfM) and compared to the degree of filamentation observed for UTI89 (WT) infection. A numerical ranking was used to categorised degree of filamentation, as summarised in the table: 1: normal rods; 2: elongated rods; 3; intermediate filaments; 4: filamentous; 5: long filaments. Estimates of the bacterial load are displayed in parentheses beside filamentation ranking. Each bacterial strain was tested on a group of 3 mice with images representative from each bladder shown. Scale bar = 50 μm. **Table A. Glycan binding profile of UTI89 rods, LEX rods and LEX filaments.** Red indicates structures that were positive for binding in all three replicates. White indicates either no binding observed, or binding was not observed in all three replicates. **Table B. Agglutination of yeast cells with bacterial cultures of 2-fold dilutions.** ^a^ Positive agglutination as determined by visual observation of yeast clumping is indicated by + and a negative result by−^b^ Fold dilution is the final dilution a positive agglutination result was achieved, n/a indicates no agglutination could be achieved with the undiluted bacterial culture. **Table C. E. coli strains and plasmids used in this study.**
(DOCX)
