# Supplementary material for: The role of bacterial size, shape and surface in macrophage engulfment of uropathogenic E. coli cells
Source: PLoS Pathog. 2024 Sep 6;20(9):e1012458. doi: 10.1371/journal.ppat.1012458 (PMC11410268; doi:10.1371/journal.ppat.1012458)
Supplement: S1 Text — Fig A. Phase contrast images of rods and filaments induced by cephalexin, ciprofloxacin and ftsZ-yfp expression. (A-D) UTI89 cells were untreated (rods) or treated with 10 μg/ml cephalexin (LEX filaments) or 15 ng/ml ciprofloxacin (CIP filaments). (E-F) UTI89/pLau80 cells were treated with 0.2% (v/v) glucose (FtsZ rods) or 0.2% (v/v) arabinose (FtsZ filaments). Samples were collected and fixed with 3.7% (v/v) formaldehyde. (A-F) Phase contrast images were acquired and cell lengths measured using a Zeiss Axioplan 2 microscope with the 100X oil immersion NA 1.4 objective. Representative phase contrast images of (A) UTI89 rods without cephalexin, (B) UTI89 LEX filaments, (C) UTI89 rods without ciprofloxacin, (D) UTI89 CIP filaments, (E) FtsZ rods, and (F) FtsZ filaments. White text in bottom left corner of images indicates average lengths of the populations. Data are from 2 independent experiments with n = 110–347. Scale bar = 5 μm. Fig B. Membrane permeability of rods and filaments induced by cephalexin, ciprofloxacin and ftsZ-yfp expression. UTI89 was treated with either cephalexin (rods: untreated, LEX rods: 2.5 μg/ml, LEX filaments: 10 μg/ml) or ciprofloxacin (rods: untreated, CIP rods: 3.75 ng/ml, CIP filaments: 15 ng/ml). UTI89/pLau80 was treated with 0.2% glucose (FtsZ rods) or 0.2% arabinose to induce expression of ftsZ-yfp (FtsZ filaments). Cells were stained with SYTO 9 and propidium iodide from LIVE/DEAD BacLight Bacterial Viability Kit, and phase contrast and fluorescence images were acquired and analysed using a Zeiss Axioplan 2 microscope with the 100X oil immersion NA 1.4 objective. (A-C) Percentage of the bacterial populations with non-permeable membranes as quantified by microscopy. Data are averages of 2 independent experiments with error bars representing the SEM, n = 131–191. Fig C. Metabolic viability of rods and filaments induced by cephalexin, ciprofloxacin and ftsZ-yfp expression. UTI89 was treated with either cephalexin (rods: untreated, LEX ro [file ppat.1012458.s001.docx]

**Supporting information**

The role of bacterial size, shape and surface in macrophage engulfment of uropathogenic *E. coli* cells

Elizabeth Peterson^1^, Bill Söderström^1^, Nienke Prins^2^, Giang H.B. Le^3^, Lauren E. Hartley-Tassell^4^, Chris Evenhuis^1^, Rasmus Birkholm Grønnemose^5^, Thomas Emil Andersen^5^, Jakob Møller-Jensen^6^, Gregory Iosifidis^1^, Iain G. Duggin^1^, Bernadette Saunders^3^, Elizabeth J. Harry^1^, Amy L. Bottomley^1^*.

**Supplementary Results
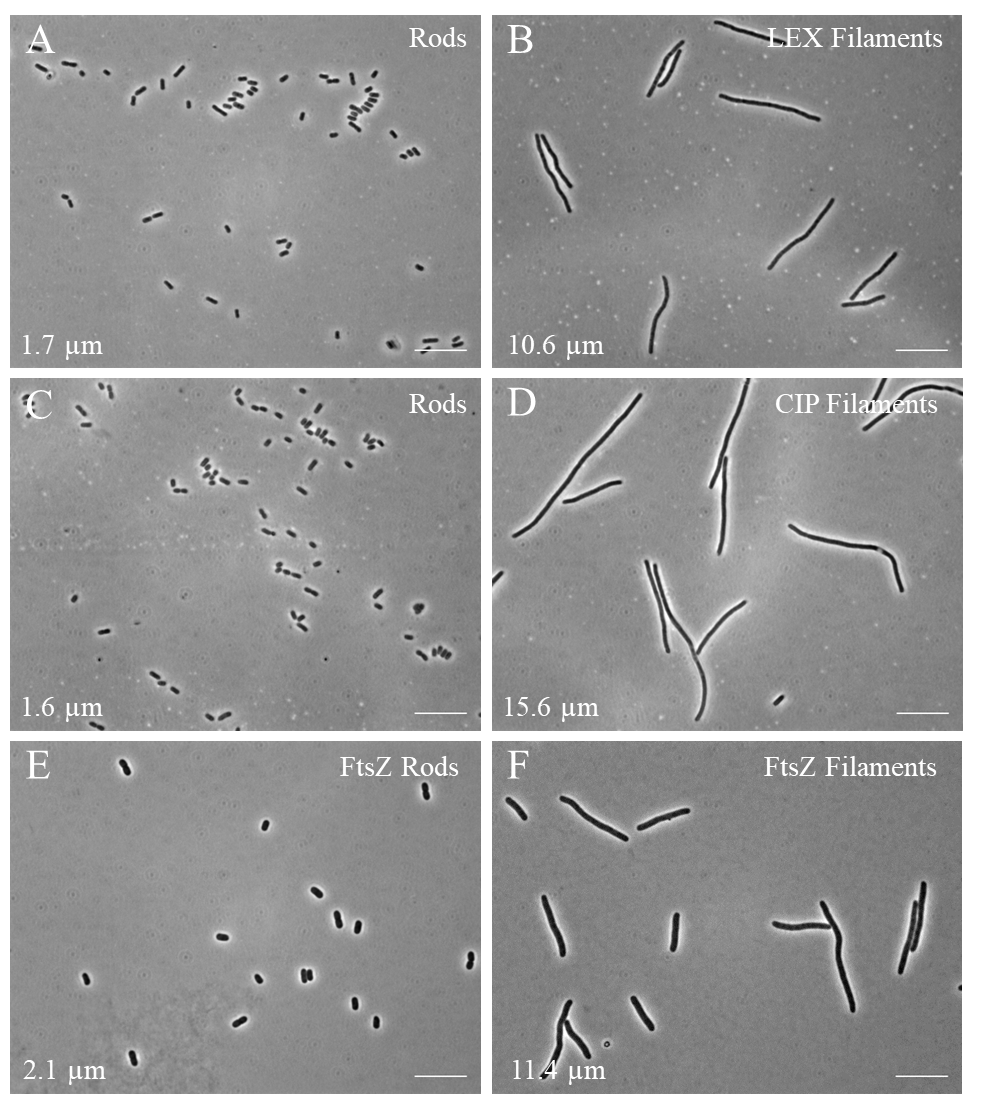
**

***Fig A. Phase contrast images of rods and filaments induced by cephalexin, ciprofloxacin and* ftsZ-yfp *expression.*** *(A-D) UTI89 cells were untreated (rods) or treated with 10 µg/ml cephalexin (LEX filaments) or 15 ng/ml ciprofloxacin (CIP filaments). (E-F) UTI89/pLau80 cells were treated with 0.2% (v/v) glucose (FtsZ rods) or 0.2% (v/v) arabinose (FtsZ filaments). Samples were collected and fixed with 3.7% (v/v) formaldehyde. (A-F) Phase contrast images were acquired and cell lengths measured using a Zeiss Axioplan 2 microscope with the 100X oil immersion NA 1.4 objective. Representative phase contrast images of (A) UTI89 rods without cephalexin, (B) UTI89 LEX filaments, (C) UTI89 rods without ciprofloxacin, (D) UTI89 CIP filaments, (E) FtsZ (plasmid repressed) rods, and (F) FtsZ-overproduction filaments. White text in bottom left corner of images indicates average lengths of the populations. Data are from 2 independent experiments with n = 110-347. Scale bar = 5 µm.*

**
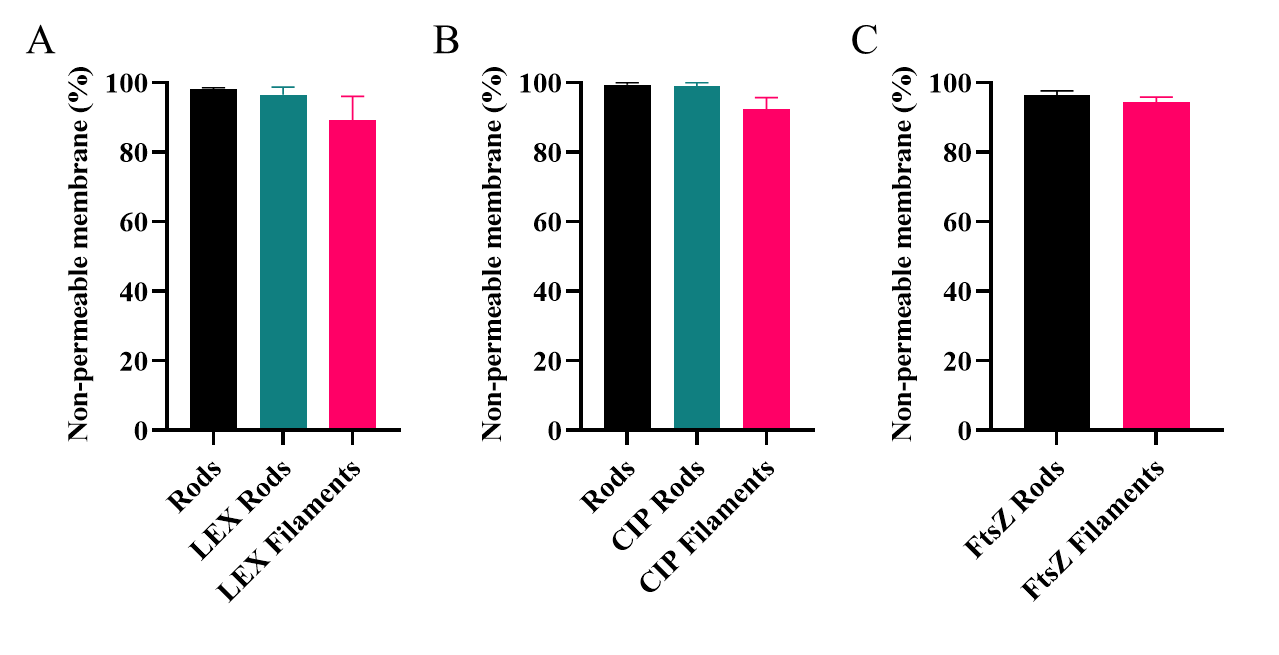
**

**Fig B. Membrane permeability of rods and filaments induced by cephalexin, ciprofloxacin and ftsZ-yfp expression.** UTI89 was treated with either cephalexin (rods: untreated, LEX rods: 2.5 µg/ml, LEX filaments: 10 µg/ml) or ciprofloxacin (rods: untreated, CIP rods: 3.75 ng/ml, CIP filaments: 15 ng/ml). UTI89/pLau80 was treated with 0.2% glucose (FtsZ (plasmid repressed) rods) or 0.2% arabinose to induce expression of ftsZ-yfp (FtsZ-overproduction filaments). Cells were stained with SYTO™ 9 and propidium iodide from LIVE/DEAD™ BacLight™ Bacterial Viability Kit, and phase contrast and fluorescence images were acquired and analysed using a Zeiss Axioplan 2 microscope with the 100X oil immersion NA 1.4 objective. (A-C) Percentage of the bacterial populations with non-permeable membranes as quantified by microscopy. Data are averages of 2 independent experiments with error bars representing the SEM, n = 131-191.


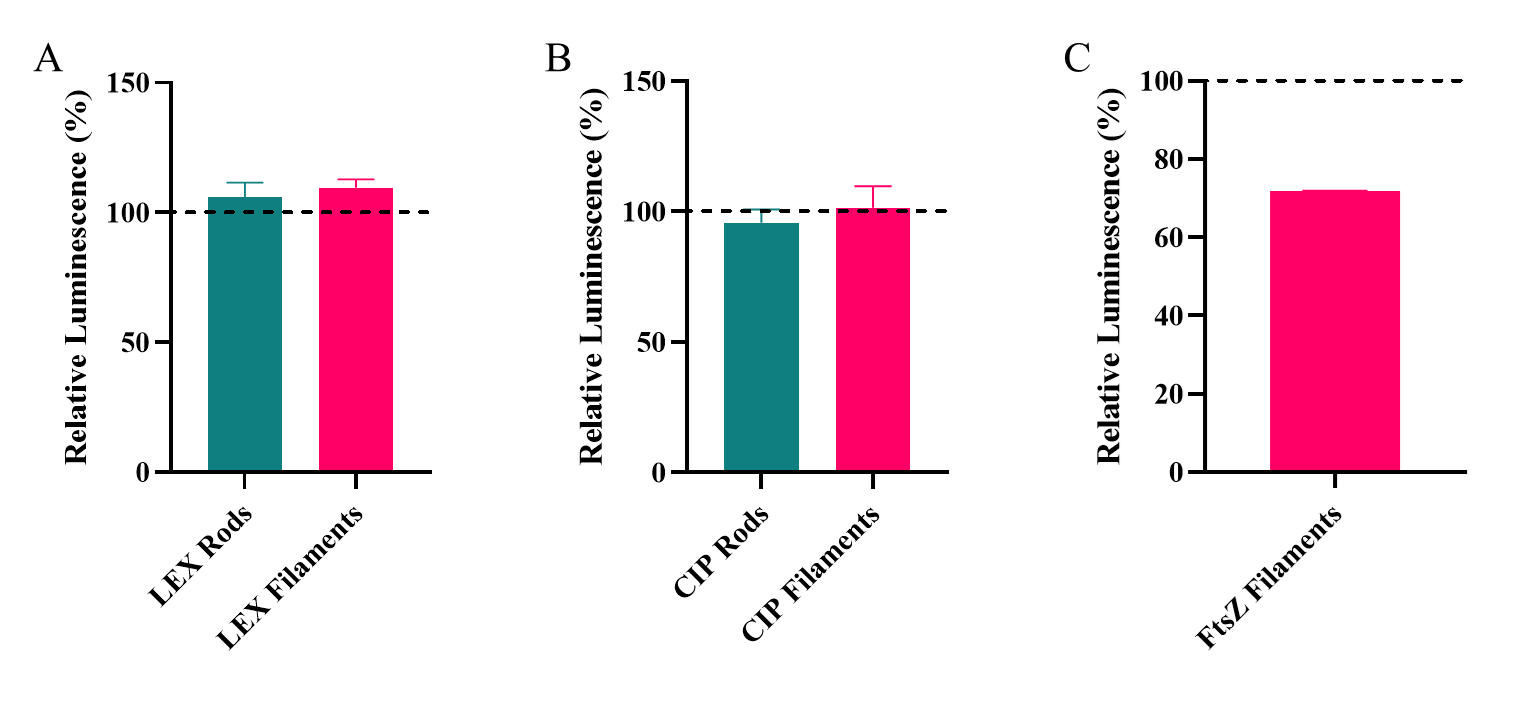


***Fig C.*** ***Metabolic viability of rods and filaments induced by cephalexin, ciprofloxacin and* ftsZ-yfp *expression.*** *UTI89 was treated with either cephalexin (rods: untreated, LEX rods: 2.5 µg/ml, LEX filaments: 10 µg/ml) or ciprofloxacin (rods: untreated, CIP rods: 3.75 ng/ml, CIP filaments: 15 ng/ml). UTI89/pLau80 was treated with 0.2% glucose (FtsZ (plasmid repressed) rods) or 0.2% arabinose to induce expression of ftsZ-yfp (FtsZ-overproduction filaments). Cells were stained with BacTiter-Glo™ Reagent from BacTiter-Glo™ Microbial Cell Viability Assay. ATP luminescence was quantified by the Tecan plate reader M200. (A-C) The relative luminescence was calculated as a percentage of the control ‘Rod’ populations (A-B: untreated rods, C: FtsZ (plasmid repressed) rods) with the dashed line representing these populations at 100%. Data are the averages of 2 independent experiments with error bars representing the SEM.*


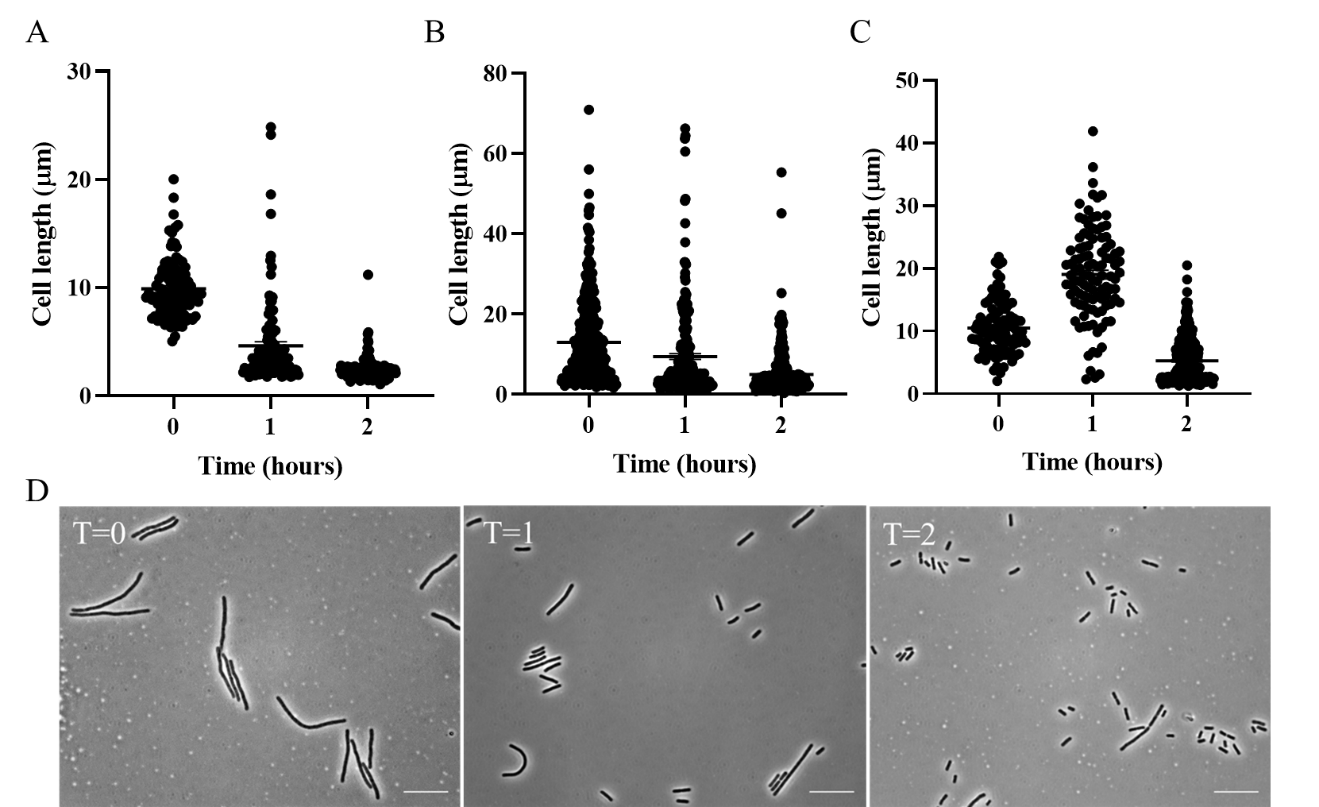


***Fig D. Reversion of UTI89 filamentous populations occurs on removal of cephalexin, ciprofloxacin or FtsZ-YFP inducer.*** *After induction of filamentation with 10 ug/ml cephalexin, 15 ng/ml ciprofloxacin or 0.2% (v/v) arabinose for FtsZ-YFP expression, cultures were centrifuged and diluted into fresh LB and incubated at 37°C. Fixed samples were taken for cell length measurement using phase contrast microscopy with Zeiss Axioplan2 using the 100X oil immersion NA 1.4 objective. (A-C) Cell length distributions over time following the removal of (A) cephalexin, (B) ciprofloxacin, or (C) 0.2% (v/v) arabinose. Data are from 2 independent experiments, n =102-355. (D) Phase contrast images showing reversion of filaments to rods over time. T indicates hours after cephalexin removal. Images are representative from 1 experiment. Scale bar = 5 µm.*


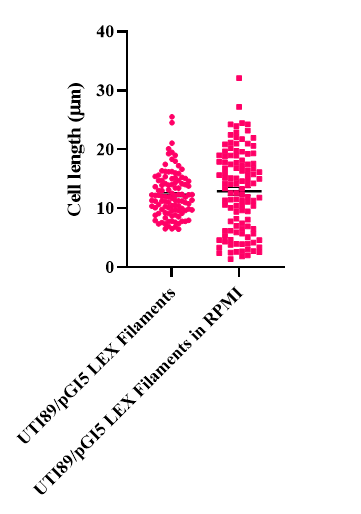


***Fig E. UTI89/pGI5 (msfGFP) LEX filaments do not revert significantly to rods during a 1-hour incubation in RPMI culture media.*** *UTI89/pGI5 (msfGFP) cells were treated with 10 µg/ml cephalexin (LEX filaments; before redilution in RPMI). To replicate gentmaicin-protection assay conditions cultures were rediluted in RPMI culture media without cephalexin for 1 hour at 37°C 5% CO_2_ (LEX filaments in RPMI). Samples were collected and fixed with 3.7% (v/v) formaldehyde. Phase contrast images were acquired and cell lengths measured using a Zeiss Axioplan 2 microscope with the 100X oil immersion NA 1.4 objective. Scatterplot of UTI89/pGI5 (msfGFP) cell length distributions are from 2 independent experiments with n = 109-113. Horizontal lines indicate mean.*

**
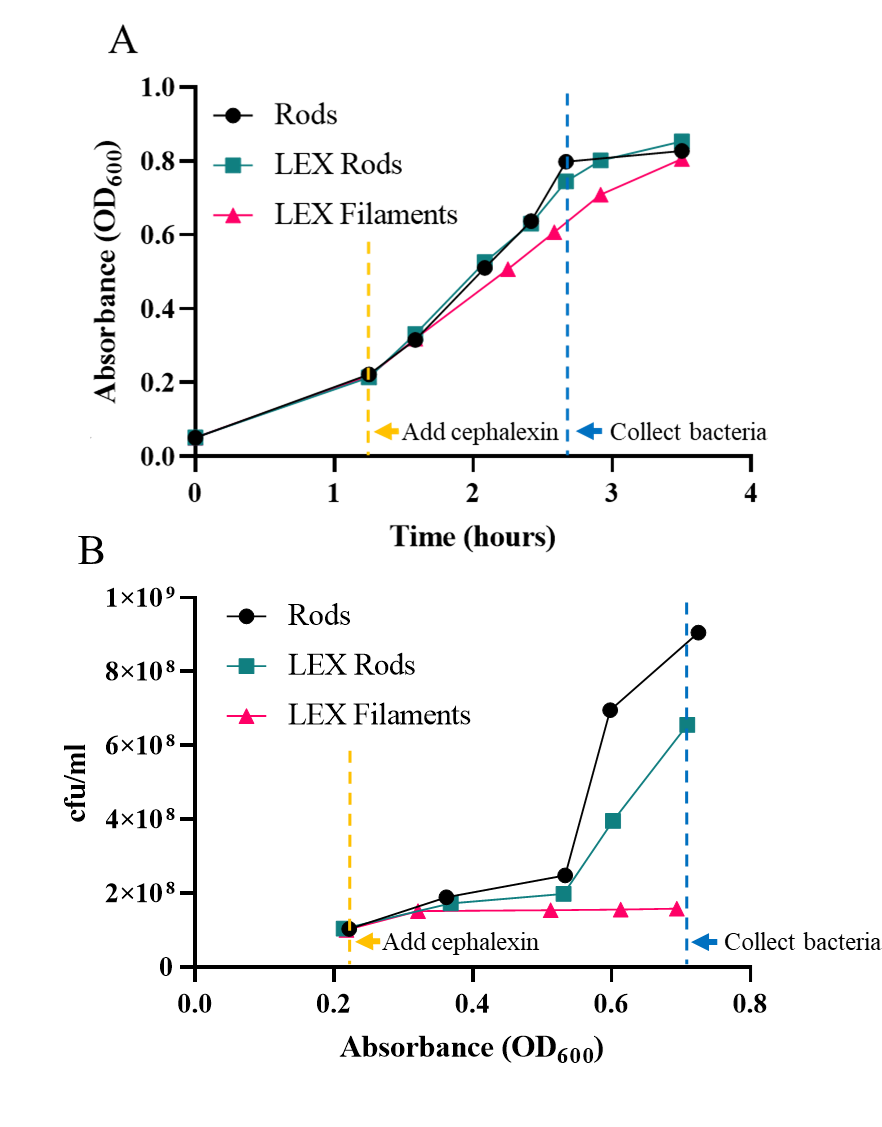
**

**Fig F.** **The relationship between cell concentration (cfu/mL) and biomass (OD_600 nm_) in cultures containing rods and filamentous cells.** UTI89 cultures were either untreated (rods), or treated with 2.5 µg/ml cephalexin (LEX rods) or with 10 µg/ml cephalexin (LEX filaments). Samples were collected over time for OD_600_ and cfu/ml measurements. The yellow dashed line indicates the time of addition of cephalexin (in LEX rods and LEX filaments cultures), and the blue dashed line indicates the time when cells were collected for determining cfu/mL and for length/volume/viability analysis and gentamicin protection assays in this study. (A) The biomass of the three populations over the growth and treatment period. (B) The number of cfu/mL in the three cultures relative to the biomass of the populations (absorbance). Data are from 2 independent experiments, with graphs representative of 1 independent experiment. After addition of 10 µg/ml cephalexin (LEX filaments), the biomass (cell growth) continues to increase, but the number of cfu/mL does not, showing that cell division but not growth has been arrested in these cultures to form the filaments observed. This indicates that as rods elongate into filaments they remain viable over time; one filament results in one cfu on average.


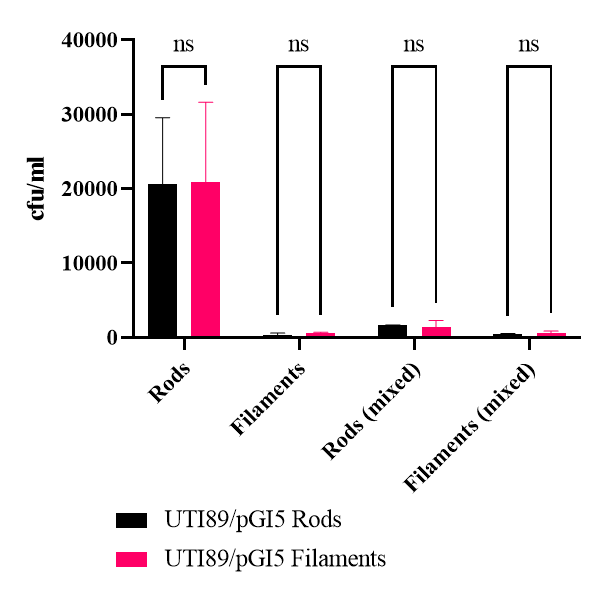


***Fig G. UTI89 containing pGI5 showed no difference in macrophage engulfment in either rod or LEX filament populations.*** *UTI89 cells were grown as separate rod or LEX filament populations before combining to produce a heterogeneous population. One population contained pGI5 (rods; black bars or filaments; red bars) to allow selective antibiotic plating. There was no significant difference in a gentamicin protection assay if wither rod or filament populations contained the plasmid as either a homogenous (rod or filament) or heterogenous (mixed) population. Data are the averages of 4 independent experiments (A and B) or 2 independent experiments (C) with error bars representing the SEM. Statistical significance determined by one-way ANOVA with multiple comparisons or Welch’s t-test.*

**Table A. Glycan binding profile of UTI89 rods, LEX rods and LEX filaments.** **
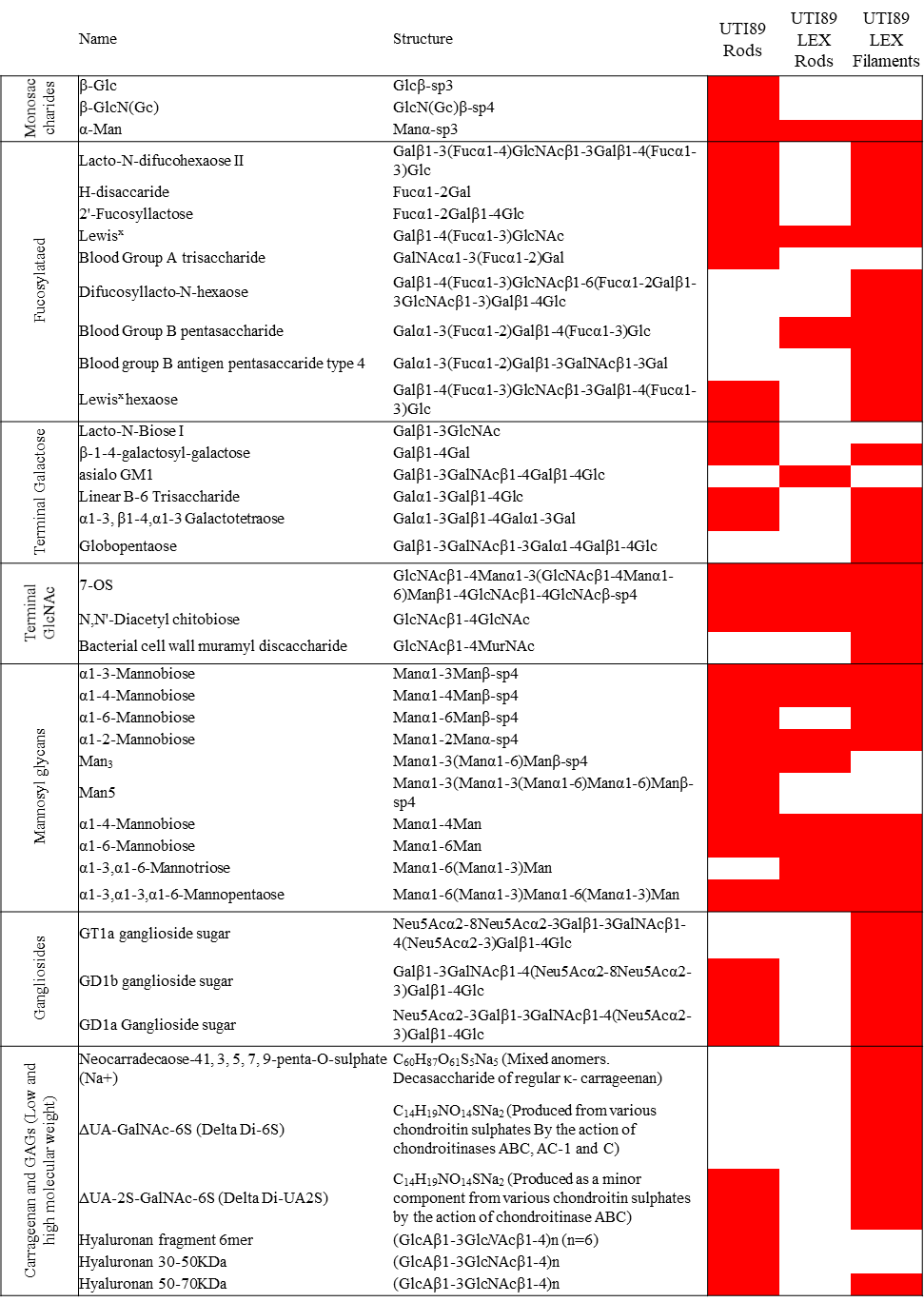
**

*Red indicates structures that were positive for binding in all three replicates. White indicates either no binding observed, or binding was not observed in all three replicates.*

**Mannose binding as determined through yeast agglutination**

The surfaces of yeast cells are rich in mannosylated glycoproteins similar to that of the mannosylated macrophage cell surface and thus, the ability of *E. coli* to bind and aggregate these yeast cells is commonly used as a proxy for functional type 1 fimbrial expression. We performed yeast agglutination assays with UTI89 rods and filaments (cephalexin-induced), and a *fimH*-deleted mutant (UTI89Δ*fimH*), to test for differences in functional type 1 fimbrial expression. We found no differences between UTI89 rod and UTI89 (cephalexin-induced) filaments ability to agglutinate yeast, and this agglutination was mannose dependent as it could be competitively inhibited by α-D-MP and D-mannose (Table B). As expected, the *fimH* mutant could not agglutinate yeast, due to the loss of functional type 1 fimbriae. We constructed a complementation strain (UTI89Δ*fimH/*pGEN-*fimH*) which restored the ability to agglutinate yeast, indicating the restoration of functional type 1 fimbriae. In summary, this indicates that there is no difference in mannose binding (and theoretically functional type 1 fimbrial expression) between rods and filaments, and no functional type 1 fimbrial expression in the *fimH* mutant.

**Table B. Agglutination of yeast cells with bacterial cultures of 2-fold dilutions.**

| **Bacteria** | **Yeast cell agglutination^a^** | **Fold Dilution^b^** |
| --- | --- | --- |
| UTI89 Rods | **+** | 1/16 |
| UTI89 Filaments | **+** | 1/16 |
| UTI89 Rods + α-D-MP | **-** | n/a |
| UTI89 Filaments + α-D-MP | **-** | n/a |
| UTI89 Rods + D-Mannose | **-** | n/a |
| UTI89 Filaments + D-Mannose | **-** | n/a |

*^a^ Positive agglutination as determined by visual observation of yeast clumping is indicated by + and a negative result by –*

*^b^ Fold dilution is the final dilution a positive agglutination result was achieved, n/a indicates no agglutination could be achieved with the undiluted bacterial culture.*


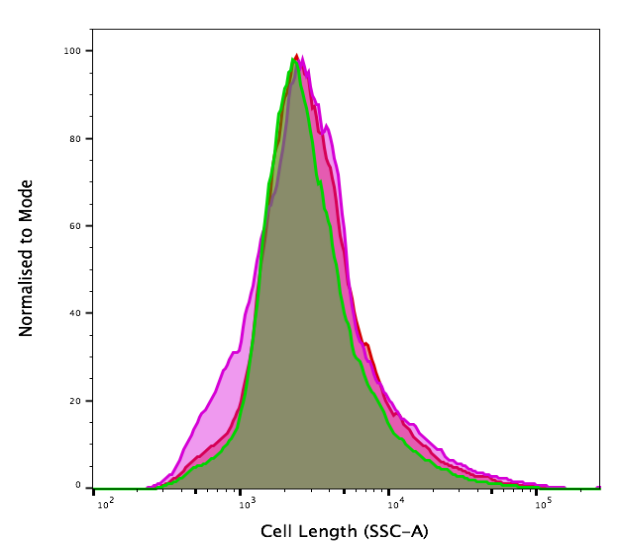

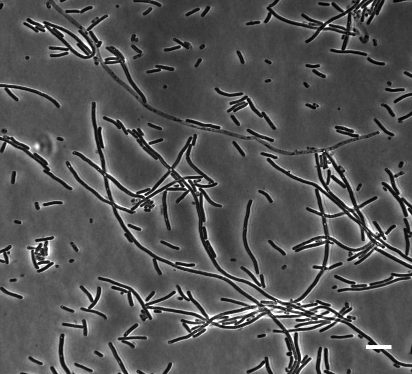

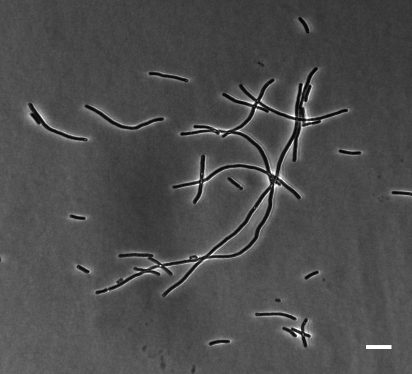

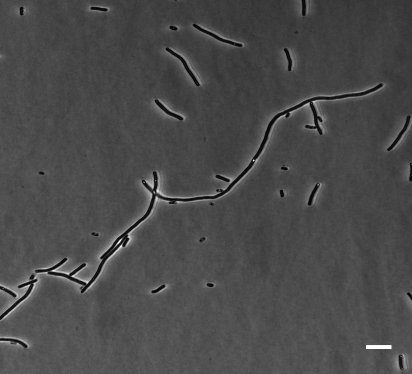


UTI89

Δ*ymfM*

Δ*sulA* Δ*ymfM*

A

C

B

***Fig H. Filamentation of bacteria isolated from the* in vitro *human bladder model.*** *(A) UTI89/pGI5 (msfGFP) were grown in and isolated from an* in vitro *human bladder model (bladder bacteria). Samples were collected and fixed with 3.7% (v/v) formaldehyde. Phase contrast images were acquired and cell lengths measured using a Zeiss Axioplan 2 microscope with the 100X oil immersion NA 1.4 objective. Cell lengths of bladder bacteria ranged from 0.72 µm to 253 µm long. Data are from 2 independent experiments with n = 337. Horizontal line indicates mean. (B) Representative phase contrast microscopy of UTI89/pGI5, ΔymfM and ΔsulAΔymfM mutants isolated from an in vitro human bladder model, showing filamentous cells for each strain. Scale bar = 5 µm (C). Flow cytometry of UTI89/pGI5 (red), ΔymfM/pGI5 (magenta) and ΔsulAΔymfM/pGI5 (green) mutants showing similar peaks indicating a bacterial population with similar morphology. The y-axis was normalised to mode to clarify the different peak positions; x-axis depicted the side scatter measurement (SSC-A) which corresponds to length of the bacteria.*


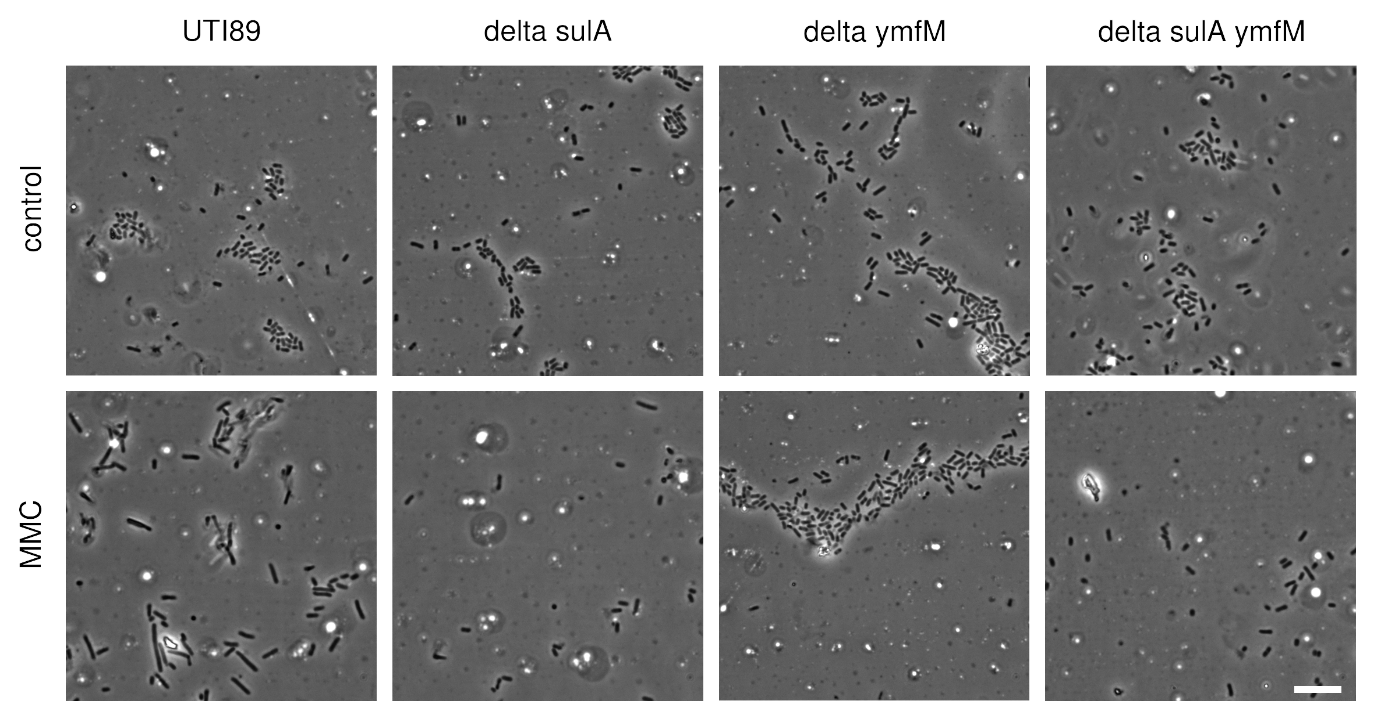

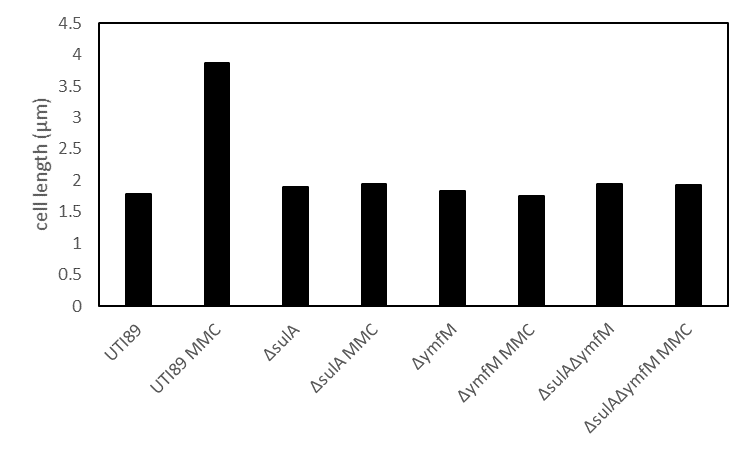


A

B

***Fig I. Filamentation of UTI89 in the presence of mitomycin C.***  *UTI89 and single sulA or ymfM mutants, and the double sulA ymfM mutant, were grown statically in LB to mid-log phase. 300 ng/ml mitomycin C was added and cells were incubated for a further 1h. Samples were observed by phase contrast microscopy (A) using a DeltaVision Elite inverted microscope with a 100x NA 1.4 objective, and average cell lengths were measured using FIJI (B). Results are from two independent experiments. Scale bar = 10µm.*

**
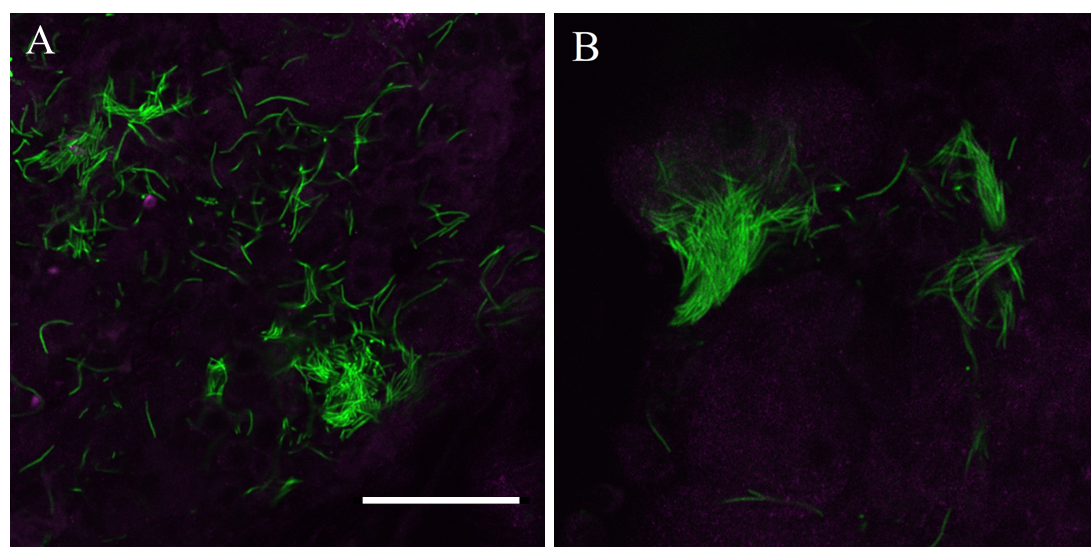
**

***Fig J. UTI89 mutants lacking sulA and ymfM retain the ability to form filaments.*** *(A-B) Six hours post-infection, with (A) UTI89ΔymfM /pMAN01 (GFP) or (B) UTI89ΔsulA/pMAN01 (GFP), C3H/HeN mouse bladders (magenta) were bisected and splayed on a silicone pad, fixed and imaged by an Olympus FV1000MPE microscope with a 20X objective (NA 0.75). Each bacterial strain was tested on a group of 4 mice with images representative from 1 mouse. Scale bar = 50 µm.*


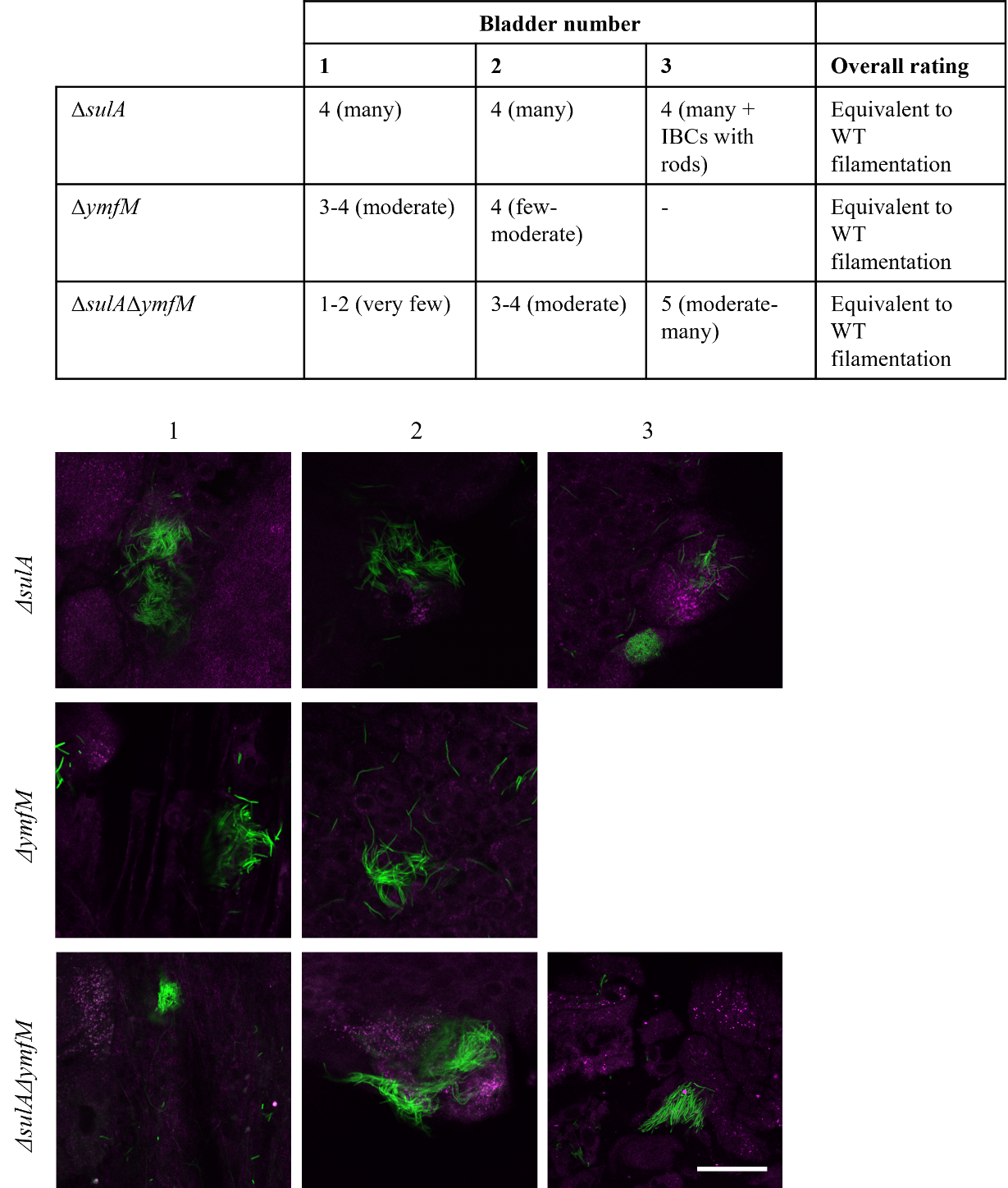


***Fig K. Filamentation of bacteria isolated from a mouse cystitis model.*** *Six hours post-infection, with UTI89Δ*ymfM */pMAN01 (GFP), UTI89ΔsulA/pMAN01 (GFP) or UTI89 Δ*sulA*Δ*ymfM */pMAN01 (GFP), C3H/HeN mouse bladders (magenta) were bisected and splayed on a silicone pad, fixed and imaged by an Olympus FV1000MPE microscope with a 20X objective (NA 0.75). Semi-quantification of degree of filamentation was assessed for 3 isolated bladders (2 for ΔymfM) and compared to the degree of filamentation observed for UTI89 (WT) infection. A numerical ranking was used to categorised degree of filamentation, as summarised in the table: 1: normal rods; 2: elongated rods; 3; intermediate filaments; 4: filamentous; 5: long filaments. Estimates of the bacterial load are displayed in parentheses beside filamentation ranking. Each bacterial strain was tested on a group of 3 mice with images representative from each bladder shown. Scale bar = 50 µm.*

**Supplementary Methods**

**Table C. E. coli strains and plasmids used in this study.**

| Plasmid or strain | Features | Reference |
| --- | --- | --- |
| pLau80 | L-arabinose-inducible promoter pBAD, *ftsZ-yfp* fusion, AMP^R^ | [1] |
| pGI5 | pSC101 origin of replication and the *Plac^Q1^* strong constitutive promotor driving expression of monomeric and and superfolder green fluorescent protein (msfGFP), SPT^R^ | [2] |
| UTI89 | O18:K1:H7 | [3] |
| UTI89/pGI5 | UTI89/pGI5 (SPT^R^) | [2] |
| UTI89/pLau80 | UTI89/pLau80 (AMP^R^) | This study |
| UTI89/pGI5/pLau80 | UTI89/pGI5/pLau80 (SPT^R^ AMP^R^) | This study |

**Viability assays**

Membrane integrity

Bacteria were cultured as described above for each of the treatments and were collected at late exponential growth phase for viability assays. Measurement of membrane permeability using the LIVE/DEAD *Bac*Light Bacterial Viability Kit (non-permeable membranes indicate viable bacteria) as per manufacturer’s instructions. Briefly, this involved staining bacteria with SYTO 9 and propidium iodide and fluorescence microscopy was used to quantify stained bacteria.

ATP production

Bacteria were cultured as described above and were collected at late exponential growth phase for viability assays. Measurement of metabolic viability by BacTiter-Glo Microbial Cell Viability Assay (based on quantitation of the ATP present where the luminescent signal is proportional to the amount of ATP present) as per manufacturer’s instructions. Briefly, this involved adding a single reagent (BacTiter-Glo Reagent) directly to bacterial cells cultured in medium and measuring luminescence. Luminescence was quantified by the Tecan Infinite M200 Plate Reader.

**Reversion of filamentous populations**

Bacterial cells were grown statically at 37°C until OD_600_=0.2-0.3, and then filamentation was induced either using 10 µg/ml cephalexin or 15 ng/ml ciprofloxacin as described in main text. UTI89/pLau80 was grown statically at 37°C for 2 hours, and then filamentation was induced using 0.2% (v/v) arabinose as described in main text. The cultures were centrifuged at 3500 *g* for 5 minutes and washed with fresh LB to remove the inducer. Cultures were then resuspended in fresh LB at OD_600_ = 0.05 and grown statically at 37°C. Samples were taken at 0, 1, 1.5 and 2 hours and then fixed with 3.7% (v/v) formaldehyde solution and prepared onto poly-L-lysine coated slides before being viewed by phase contrast microscopy.

**Glycan microarrays**

UTI89 was grown statically at 37°C until OD_600_=0.2-0.3, and then filamentation was induced using 10 µg/ml cephalexin as described. The samples were washed in PBS and then stained at room temperature with either CFDA or Bodipy-595 methyl ester lipophilic dye for 30 minutes then fixed in 3.7% (v/v) formaldehyde, before being made up at 1 x 10^8^ CFU/ml. Glycan arrays were printed as previously described by Waespy, Gbem (94), Day, Tiralongo (95). Briefly, 395 glycans were printed at a concentration of 500 µM using an Arrayit SpotBot Extreme protein edition array printer onto SuperEpoxy3 microarray slides (Arrayit). Glycans were printed using solid 946MP4 printing pins. After the printed slides were neutralised, the slides were blocked for 5 minutes in 0.5% (v/v) BSA in PBS. The slides were then rinsed in PBS and dried by centrifugation at 200 *g* for 4 mins. For each slide, a 65 µL geneframe was placed on the slide, and 65uL of the 1 x 10^8^ CFU/ml cells in array PBS were added to the slide and a coverslip was applied to the geneframe. The slides were then incubated for 30 minutes at room temperature in the dark. After the incubation time, the slide was then immersed in array PBS and washed briefly for two minutes with gentle shaking. The array PBS was replaced, and the slide was washed gently for an additional 2 mins. The slide was then transferred to a clean 50 ml tube, to dry by centrifugation at 200 *g* for 4 mins.

The slide was then scanned using an Innopsys Innoscan 1100AL microarray scanner, using the 488 nm, 532 nm and 635 nm lasers, using low laser power and 70% PMT gain settings. The acquired image was then analysed using the Mapix software, overlaying the image with the map (Gal) file. The experiment was repeated three times, and binding was classified as positive when the average RFU (relative fluorescence units) of a specific structure had a value above mean background (defined as the average background fluorescence plus 3 standard deviations) and had a *P*-value of < 0.005 (student’s T-Test). A pre-experimental image was acquired to rule out any autofluorescence of the printed glycans.

**Yeast agglutination assay**

Performed as described by Crépin, Lamarche (96) with the following additions. A single colony of the desired strains were inoculated in 5 ml LB at 37°C and grown statically overnight. Cultures were diluted into fresh LB, grown and induced to form filaments as described by addition of cephalexin. Cultures were centrifuged at 4000g for 5 minutes and resuspended in either PBS, in PBS with 3% (w/v) D-Mannose or 3% (w/v) methyl α-D-mannopyranoside. Assays were then performed as described by Bottomley, Peterson (64). Briefly, cultures were serially diluted 2-fold before 100 µl of each sample was added into 24 well plates. 20 µl of 3% (w/v) bakers yeast suspension was then added to each well. Yeast aggregation was observed visually and the agglutination titre of the most diluted bacterial sample giving a positive agglutination reaction (defined by clumps forming in a clear solution) at room temperature after 10 minutes was recorded. Technical and biological replicates were performed twice.

**Reference**

1. Lau IF, Filipe SR, Søballe B, Økstad O-A, Barre F-X, Sherratt DJ. Spatial and temporal organization of replicating Escherichia coli chromosomes. Molecular Microbiology. 2003;49(3):731-43. doi: <https://doi.org/10.1046/j.1365-2958.2003.03640.x>.

2. Iosifidis G, Duggin IG. Distinct Morphological Fates of Uropathogenic Escherichia coli Intracellular Bacterial Communities: Dependency on Urine Composition and pH. Infect Immun. 2020;88(9). doi: 10.1128/IAI.00884-19. PubMed PMID: 32540870; PubMed Central PMCID: PMCPMC7440767.

3. Mulvey, M. A., J. D. Schilling and S. J. Hultgren (2001). "Establishment of a persistent *Escherichia coli* reservoir during the acute phase of a bladder infection." Infection and immunity **69**(7): 4572-4579.
